# The relationship between regular substance use and cost comparisons in stable and volatile learning contexts

**DOI:** 10.1038/s41398-026-03830-z

**Published:** 2026-01-30

**Authors:** Sonia G. Ruiz, Samuel Paskewitz, Arielle Baskin-Sommers

**Affiliations:** Department of Psychology, Yale University, New Haven, CT USA

**Keywords:** Human behaviour, Addiction

## Abstract

Insensitivity to costs during cost-benefit decision-making consistently has been related to substance use severity. However, little work has manipulated cost information to examine how people evaluate and compare multiple costs. Further, no work has examined how the consideration of cost information varies across different contexts. We administered a new loss-frame variant of a probabilistic learning task in a diverse community sample enriched for substance use (*N* = 137). Individuals with more years of regular substance use tended not to repeat choices after they avoided losses, choosing similarly regardless of whether they had avoided or incurred a loss. Computational modeling parameters indicated that they were more inconsistent in their use of expected values to guide choice. These results contribute to our conceptualization of substance use severity by suggesting that inconsistency in using cost information, rather than insensitivity to costs, may inform choices to continue using substances despite incurring negative consequences.

## Introduction

A hallmark of substance use severity is the tendency to continue use despite negative social, legal, financial, or health consequences [1, 2]. Some people continue using substances despite experiencing strained family relationships, debt, child custody issues, or cardiac or respiratory problems. Often, these consequences are embedded within uncertain contexts, such as inconsistent healthcare, unreliable physical and social support [3, 4], and unemployment [5, 6], that exacerbate the desire for and use of substances. However, for some people, there also are negative consequences for not using substances. Qualitative research points to loneliness [7], difficulty using new coping strategies [8], loss of identity [9], and withdrawal symptoms [10] as powerful reasons why not continuing to use substances is costly. The consequences of not using substances also may vary by context [11]. Thus, inherent in the severity of substance use is a comparison of different costs that come from using (e.g., if I use, my family will cut me off) and behavioral change (e.g., if I stop, I’ll be lonely because all my friends use) that may depend on context (e.g., when social support is unreliable).

Within the substance use literature, some theoretical work considers cost comparison within the relative utility of use vs. non-use (e.g., Herrnstein-Prelec theory [12]; Becker-Murphy theory; relative addiction theory [13]; incentive sensitization [14], temporal myopia [15]; predictive processing [16]). In experimental research, a decision-making framework has long been used to study how people consider benefits and/or costs [17]. Paradigms used to test decision-making typically do so using rewards only, single costs, or single contexts [18]. First, most paradigms focus on choices made to obtain rewards. In these paradigms, people choose among rewards associated with different delays (e.g., smaller sooner reward vs. larger later reward), effort levels (e.g., smaller, less-effortful reward vs. larger, more-effortful reward), risks (e.g., smaller, more certain reward vs. larger, uncertain reward), and contingencies (e.g., the more frequently rewarded option changes). Greater substance use severity is related to increased sensitivity to delay in rewards [19], effort to obtain rewards [20], decreased sensitivity to the risk of an uncertain reward [21–23] (c.f., [24–26]), and perseveration on a previously rewarded option [27]. These reward-focused paradigms ultimately only include rewards or omitted rewards as outcomes.

Second, some paradigms focus on choices in the context of costs. In these paradigms, people choose among options associated with different feedback (e.g., comparing outcomes of chosen vs. unchosen option) and risks of costs (e.g., comparing options that more frequently incurred vs. less frequently incurred loss). Greater substance use severity is linked to insensitivity to relative cost feedback [28], insensitivity to cost feedback [29], and perseveration after incurring costs [30]. These cost-focused paradigms only present a single cost that people must learn to avoid.

Finally, most paradigms only test choices in a single context, often where the only source of uncertainty is the probabilistic relationship, or contingency, between choice and outcome. For example, in contexts where contingencies remain consistent across the task, people with greater substance use severity generally choose larger rewards that lead to net losses [31], riskier rewards [32], do not integrate unexpected outcomes, and perseverate on no-longer rewarded choices, thereby incurring costs [33, 34]. However, not much is known about how people with greater substance use severity make choices in different contexts, particularly those that feature multiple sources of uncertainty. Decision-making studies conducted not involving substance use find that people make more variable and costlier choices in contexts where contingencies frequently change (i.e., are volatile) than in contexts where contingencies remain consistent (i.e., are stable) [35]. Volatility contributes to uncertainty regarding whether unexpected outcomes signal change in contingencies, and how quickly those outcomes are integrated to inform choices.

Altogether, while much has been learned about the relationship between substance use severity and decision-making around benefits and/or costs, no paradigms test how substance use severity relates to choices among multiple potential costs, across different contexts. As a result, we have a limited understanding of how individuals with greater substance use severity compare costs when making choices and which contexts may increase their susceptibility to costlier choices. Manipulating cost information is important for understanding substance use severity because substance use often occurs in a complex decision environment that necessitates a cost comparison. For example, the substance use literature often describes the tension between avoiding negative affect with substance use at the cost of well-being versus exerting the effort to learn a new strategy for managing emotions [13–16]. Further, this tension may change based on context, such as when someone is in an unfamiliar setting versus when stably connected to community resources. Clarifying *how* individuals make cost comparisons may better explain *which* costs matter (e.g., loneliness, negative emotions, withdrawal symptoms) and *when* (i.e., related to specific situations).

The goal of the present study was to assess how substance use severity relates to the consideration and comparison of cost information in different contexts. Substance use severity has been defined in many ways: for example, number of symptoms (e.g., DSM-5 specifier) [19, 20, 36], clinical impairment (e.g., substance use disorder diagnosis) [23, 27], use history [29], use amount (e.g., number cigarettes/day [19]), and use frequency [37–40] (e.g., percent heavy drinking days [41]; chronic use [34]; weekly use [19]; regular use [42–44]). One indicator of severity, which also is associated with number of symptoms, impairment, use history, frequency, and amount, is years of regular use [42–45]. In the present study, we used a modified version of the ASI [44], the ASI-X [42, 43, 45], that focuses on assessing the cumulative years of regular use, as the primary indicator of severity. We administered a novel loss-frame variant of a widely used probabilistic learning task [46] with a stable and volatile context. First, we examined choice behavior via regression to determine how cost feedback informed behavior (i.e., stay vs. shift choices) across each context. Second, to assess learning from cost information, we applied computational models (e.g., Rescorla-Wagner, Sutton-K1, Hierarchical Gaussian Filter). Our analyses allowed us to not only examine behavior overall (i.e., stay choices given cost feedback), but also to characterize how individuals arrived at their choices (i.e., via cost sensitivity, uncertainty about outcomes).

Consistent with prior reward-based probabilistic learning research, we hypothesized that greater variability in stay choices would be seen in the volatile context [46–48], and more years of regular substance use would be associated with more stay choices (i.e., perseveration) following incurred losses [21], especially in the volatile context given descriptive research on substance use showing sensitivity to uncertainty [49]. Because computational modeling analyses were exploratory and primarily used to interpret choice behavior, we did not have specific hypotheses about relationships between individual parameters and outcomes.

## Ethics approval and consent to participate

All methods were performed in accordance with the relevant guidelines and regulations. Approval was obtained from the Yale University Institutional Review Board (Protocol #1408014485). Informed consent was obtained from all participants. No live vertebrates were involved in this research.

## Materials and methods

### Participants

To represent a range of substance use severity, a non-random, self-selected sample of adults was recruited from New Haven County, Connecticut through flyers soliciting risk-taking behavior. A pre-screen phone interview and in-person assessment materials were used to exclude individuals who were younger than 18 or older than 65; performed below fourth-grade level on a standardized reading measure; scored below 70 on a standardized IQ measure; were diagnosed with schizophrenia, bipolar disorder, or psychosis, not otherwise specified or had a family history of psychosis; took antipsychotic, anticonvulsant, or mood stabilizers; or had medical problems that could impede comprehension of or performance on the task. Participants earned $15/hour and a bonus (range = $0–$10, rounded to the nearest dollar) depending on the sum of two randomly selected trials from the probabilistic learning task.

Previous studies using a probabilistic learning task detected relationships between task-derived parameters and individual difference variables using sample sizes of 31-89 participants [47, 48, 50–53]. An a priori power analysis indicated a sample size of 111 or higher would provide 80% power to obtain effect sizes similar to previous work (0.15-0.3). Using simulation to generate and test data based on the models used in the present study, a sensitivity analysis estimated power to detect a three-way interaction between two within-subjects task variables (e.g., context, previous outcome) and a between-subjects individual difference variable (e.g., substance use severity) controlling for covariates (e.g., difference in loss magnitude between the chosen and unchosen option, randomization, participant age) using a mixed effects logistic regression model [54], given a sample size of 137. At 137 participants there would be 89 and 100% power to detect two-way interactions of effect size 0.17 and 0.23, respectively.

### Measures

#### Modified addiction severity index (ASI-X)

Interviewers asked participants whether they had engaged in lifetime use of substances including alcohol, cannabis, cocaine/crack, methamphetamines, other amphetamines, heroin, other opioids, hallucinogens, inhalants, nicotine, and other drugs. If a participant endorsed use of a substance, they were asked how old they were when they initiated use. Then, they were asked about frequency of use, specifically if they engaged in regular use (three or more times weekly) or less frequent (i.e., twice weekly, once weekly) use. If they endorsed regular use (three or more times weekly), they were asked their ages at which periods of regular use started and ended, to quantify the total number of years of regular use for each substance. Finally, all participants who endorsed regular or less frequent use were asked the age at which they most recently used the substance.

Following prior research [42, 43, 45], regular use was defined as three or more times weekly. Years of regular use across substances were summed to determine lifetime regular use across substances, where scores of zero reflected no regular use of any substance and scores greater than zero reflected regular use of at least one substance in their lifetime. Note that scores of zero could overlap with less frequent use of a substance (i.e., using once weekly, which does not meet threshold for regular use).

#### Probabilistic learning task

Participants completed a new loss-frame variant of a probabilistic learning task [46], programmed in PsychoPy3 (version 2022.1.1) [55].

For each of 200 trials, participants chose between a white card and a black card with different loss magnitudes (randomized between $1 and $5). Each trial had a correct and incorrect card based on set probabilities described below (unknown to the participant), and their goal was to pick the correct card. If their chosen card was incorrect, they would lose the amount of money presented on the card; if their chosen card was correct, they would lose $0. Participants were informed that the likelihood each card was correct (i.e., its probability of loss) was not equal, such that one card had a higher or lower chance of being correct. They were instructed to build a feeling for which card was correct based on past outcomes, and to consider the chance that their selected card could be correct alongside the amount of money they could lose if that card was incorrect. Participants did not have a limit on the amount of time to make a choice. After they made their choice, the correct card with the amount of money lost (either $0 if their choice was correct, or between $1 to $5 if their choice was incorrect) was shown in the middle of the screen.

The task had two contexts: stable (where contingencies remained the same for 100 trials) and volatile (where contingencies often changed during 100 trials). Participants were not told that the task was divided into two contexts. In the stable context, one card predicted loss with a probability of 75% and the other with probability of 25% for all 100 trials. In the volatile context, one card predicted loss with a probability of 80% and the other with 20%, and the probabilities associated with each card changed every 25 trials. Task context order (stable first vs. volatile first) was randomized across participants.

**Fig. 1:**
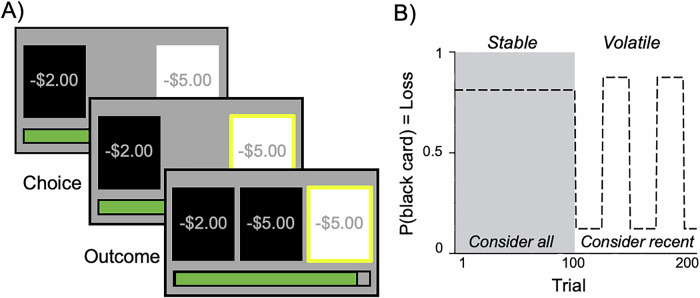
Schematic of Loss-Frame Probabilistic Learning Task. **A** Example trial of the loss-frame probabilistic learning task. On each trial, participants chose between white and black cards with loss magnitudes randomized between $1 and $5. After making a choice (highlighted in yellow), participants were shown the correct card and amount of money lost on that trial (e.g., -$5). The green bar at the bottom of the screen tracked how much money participants had lost cumulatively until the current trial. **B** Trial-by-trial probability of loss for the black card (75% in the stable context; alternating 20 and 80% every 25 trials in the volatile context, as learned by the participant).

This task aimed to assess how much participants could balance loss magnitudes with probabilities (i.e., the lower-magnitude card was not always better) and context (i.e., the same card did not always predict loss). For example, in the stable context, the probabilities associated with each card remained the same for 100 trials, suggesting that recent losses were just as important as older losses. In the volatile context, the probabilities associated with each card switched often, suggesting that recent losses should carry more weight in guiding choices (Fig. 1).

Prior to beginning the task, participants were informed that the amounts lost on two trials would be randomly selected and subtracted from a $10 bonus, which would be added onto their hourly compensation. A green bar onscreen recorded how much the participant lost cumulatively up until the current trial.

Participants completed four practice trials before the task began and were monitored via a camera by research assistants in another room to ensure proper task engagement. 141 participants completed the task. Participants were excluded if they did not complete the task (*N* = 1) or if they showed poor concentration (e.g., clicking same answer; *N* = 3). Excluded participants did not differ from included participants in terms of age (*t*(3.10) = 0.33, *p* = 0.765), sex (*X*^*2*^ (1, *N* = 141) = 10^-29^, *p* = 1.000), or ASI-X (*t*(3.28) = 0.64, *p* = 0.563). The final sample was 137 participants (Table S1).

### Data analysis

#### Choice behavior analysis

Analyses were conducted using the lme4, lmerTest, and interactions packages in R version 4.2.2 [56–59]. Two mixed-effects logistic regressions tested for (1) basic task effects and (2) the contribution of individual differences in years of regular substance use (square root-transformed, z-scored) to cost considerations [47, 60]:

1 $${Stay}\, \sim \,{Context}\times {Previous}\,{outcome}+{Loss}\,{magnitude}\,{difference}+{Age}+{Randomization}+(1{|Subject})$$

2 $${Stay}\, \sim \,{Context}\times {Previous}\,{outcome}\times ASI-X+{Loss}\,{magnitude}\,{difference}+{Age}+{Randomization}+\left(1{|Subject}\right)$$

All models controlled for the difference in loss magnitude between the chosen and unchosen option (mean-centered; given interest in specific contribution of outcomes to choice), age (mean-centered; given that age explains variability in both lifetime substance use and win-stay/lose-shift behavior [61, 62], see Figure S1), and randomization (stable first vs. volatile first; given the impact of context order on sensitivity to outcomes, see Supplemental Material and Figure S2). Models included a random intercept for each subject.

Significant interactions were followed up on with simple slopes analyses [63]. Supplemental Material presents additional analyses testing basic task checks, replicating basic task effects and effects related to anxiety/stress, concurrent (executive functions, impulsivity) and discriminant validity (trait absorption, achievement, and social potency), as well as robustness of effects (i.e., ASI-X controlling for executive functions and impulsivity).

#### Computational modeling

While choice behavior analyses captured *overall preferences* indicated by participants’ observed choices, we also aimed to model the learning process that *generated* each choice. To estimate participants’ learning processes across stable and volatile contexts, the following models [64] were fit to participants’ choices: a Rescorla-Wagner model [65], K1 Sutton model [66], and 2- and 3-level Hierarchical Gaussian Filter (HGF) models [67, 68]. All models were fitted using the HGF toolbox version 7.1.3 (https://www.tnu.ethz.ch/en/software/tapas) [69]. Model fitting procedures were conducted in MATLAB version R2022a [70]. Table S2 displays priors. Models were compared using Bayesian model selection [71] via the Statistical Parametric Mapping 12 toolbox (https://www.fil.ion.ucl.ac.uk/spm/). Bayesian model selection uses the log-evidence from each model to determine the likelihood that a specific model generated the observed choice data for a randomly chosen participant, and the likelihood of one model being more frequent than all other models across all participants (i.e., exceedance probability).

**Fig. 2:**
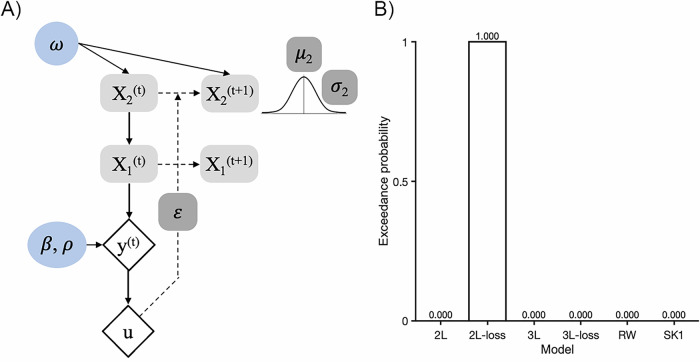
Schematic of the Two-level Hierarchical Gaussian Filter with Loss Sensitivity. **A** Diagram of 2-level HGF with loss aversion. Volatility parameter *ω* modulates the speed of belief updates about choice (*y*) -outcome (*u*) contingencies. The SoftMax observation model transforms predicted probabilities of a positive outcome associated with card 1 on trial *t*, modulated by inverse temperature β and loss aversion ρ, into choice *y*. **B** Model comparison results. 2 L = 2-level HGF; 2L-loss = 2-level HGF with loss aversion parameter; 3 L = 3-level HGF; 3L-loss = 3-level HGF with loss aversion parameter; RW = Rescorla-Wagner; K1 = Sutton K1 model.

Broadly, the HGF describes how individuals learn in uncertain environments. This model estimates learning as a hierarchical process, where learning about trial-by-trial choice-outcome contingencies (i.e., which card is more likely to lose) is a function of learning about characteristics of the environment, such as how quickly or how often its contingencies change (Fig. 2a). Participants’ choices *y* and outcomes on each trial *u* were used to estimate the following parameters: (1) volatility ω, or how quickly people update their beliefs, or learn, about choice-outcome contingencies (where higher values reflect faster updating), (2) inverse temperature β, or how consistently people choose the better expected value (where higher values reflect greater consistency), and (3) loss aversion ρ, or how sensitive people are to loss magnitudes (where higher values reflect greater loss sensitivity). These parameters influence trial-by-trial trajectories comprised of beliefs about choice-outcome contingencies (e.g., how likely each card is to lose; μ_2_), uncertainty about those contingencies (σ_2_), and surprise at outcomes (ε_2_). Trajectories, in turn, influence predictions about outcomes on each trial (e.g., choosing card 1 will result in an avoided loss). For model equations, see Supplemental Material.

To assess how regular substance use contributed to learning processes, three linear regressions with each 2-level HGF loss aversion model parameter (volatility ω, log-transformed inverse temperature β, and log-transformed loss aversion ρ) as the continuous outcome tested for relationships with years of regular substance use (square-root transformed and z-scored).

3 $${Parameter} \sim ASI-X+{Age}+{Randomization}$$

One extreme outlier for inverse temperature β was winsorized to within one standard deviation of the maximum non-extreme outlier value. All analyses controlled for randomization (stable first vs. volatile first) and age (mean-centered). Supplemental Material presents additional validity and robustness analyses.

## Results

### Variable descriptives

Correlations across sample demographics, individual difference variables, choice behavior, and modeling parameters are provided in Figure S1. Distributions of individual difference variables (Figure S3), choice behavior (Figure S4), and task validity checks (Figures S5-S8) are provided in the Supplemental Material. Sample descriptives for regular substance use appear in Table S2. 75% (*n* = 103) of the sample engaged in regular use of at least one substance in their lifetime and 97% of the sample endorsed use less frequent than three times weekly (Table S3).

### Choice behavior analysis

#### Basic task effects

**Fig. 3:**
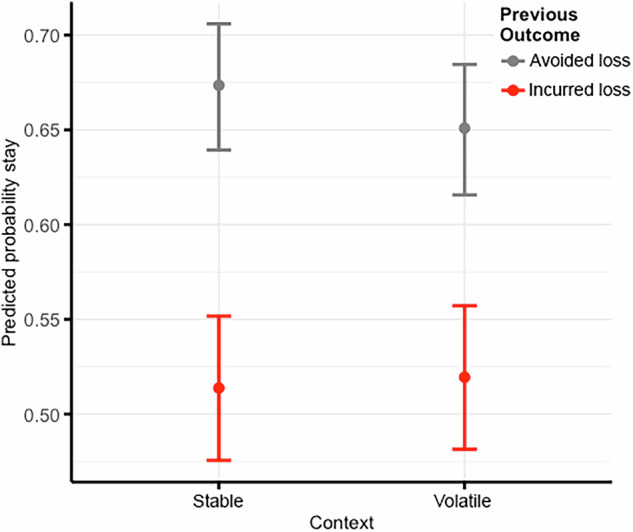
Previous Avoided Loss and Stable Context Predict Stay Choices. Error bars represent 95% confidence intervals for point estimates.

As hypothesized, results from the basic task effects regression (Eq. 1) indicated that participants were more likely to stay on the same option after an avoided loss in the stable context, suggesting that they adjusted choices according to the task context manipulation (Table 1; Fig. 3). Supplemental Material includes additional tests of choices as a function of different task variables (i.e., choice proportion expected value, lower magnitude loss, left option (indexing random choices), stayed on the same chosen option, stayed on the same chosen option after avoided loss, and stayed on the same chosen option after incurred loss; trial proportion of incurred losses; total summed loss; and mean reaction time; see Figure S4), analyses replicating basic task effects found in other versions of the probabilistic learning task (i.e., choice proportion best expected value; choice proportion lower probability of loss; choice proportion lower magnitude loss; choice proportion stayed after avoided loss compared to after incurred loss across task contexts; see Figure S5), and validity checks (i.e., anxiety/stress effects [Tables S4 and S5]; concurrent and discriminant validity checks [Tables S4 and S5; Figure S8]).

**Table 1:** Choice Behavior and Computational Modeling Regression Results.

Equation	Model terms of interest	β	*SE*	95% CI	*z or t*	*p*
1	Previous outcome	−0.669	0.037	−0.741, −0.597	−18.175	10^−16^
	Context	−0.101	0.036	−0.172, −0.031	−2.816	0.005
	Previous outcome × Context	0.124	0.052	0.023, 0.225	2.397	0.017
	Avoided loss vs. Incurred loss					
	Stable *OR*	1.950	0.072	1.820, 2.100	18.174	0.001
	Volatile *OR*	1.720	0.063	1.610, 1.850	14.978	0.001
	Stable vs. Volatile					
	Avoided loss *OR*	1.110	0.040	1.031, 1.190	2.816	0.005
	Incurred loss *OR*	0.977	0.036	0.909, 1.050	−0.613	0.540
2	Previous outcome × ASI-X	0.226	0.038	0.152, 0.300	6.015	10^−9^
	Avoided loss: ASI-X slope	−0.230	0.060	−0.347, −0.113	−3.845	10^−4^
	Incurred loss: ASI-X slope	−0.022	0.060	−0.140, 0.095	−0.370	0.711
	Context × ASI-X	0.083	0.037	0.010, 0.155	2.243	0.025
	Stable: ASI-X slope	−0.158	0.060	−0.275, −0.041	−2.638	0.008
	Volatile: ASI-X slope	−0.093	0.060	−0.211, 0.023	−1.572	0.116
	Previous outcome × Context × ASI-X	−0.038	0.053	−0.141, 0.066	−0.713	0.476
3a	ASI-X effect on volatility	0.182	0.257	−0.327, 0.690	0.707	0.481
3b	ASI-X effect on loss aversion	0.144	0.093	−0.040, 0.328	1.548	0.124
3c	ASI-X effect on inverse temperature	−0.307	0.127	−0.558, −0.056	−2.416	0.017

For reference, the equations are: (1) Stay ~ Previous outcome × Context + Loss magnitude difference + Randomization + Age + (1|Subject); (2) Stay ~ Previous outcome × Context × ASI-X + Loss magnitude difference + Randomization + Age + (1|Subject); and (3a-c) HGF model parameter ([a] volatility, [b] loss aversion, [c] inverse temperature) ~ ASI-X + Randomization + Age. Note that Eq. 1 is one model, Eq. 2 is one model, and Eqs. 3a–3c are three separate models.

*ASI-X* Modified Addiction Severity Index; *OR* Odds Ratio.

#### Considering regular substance use

**Fig. 4:**
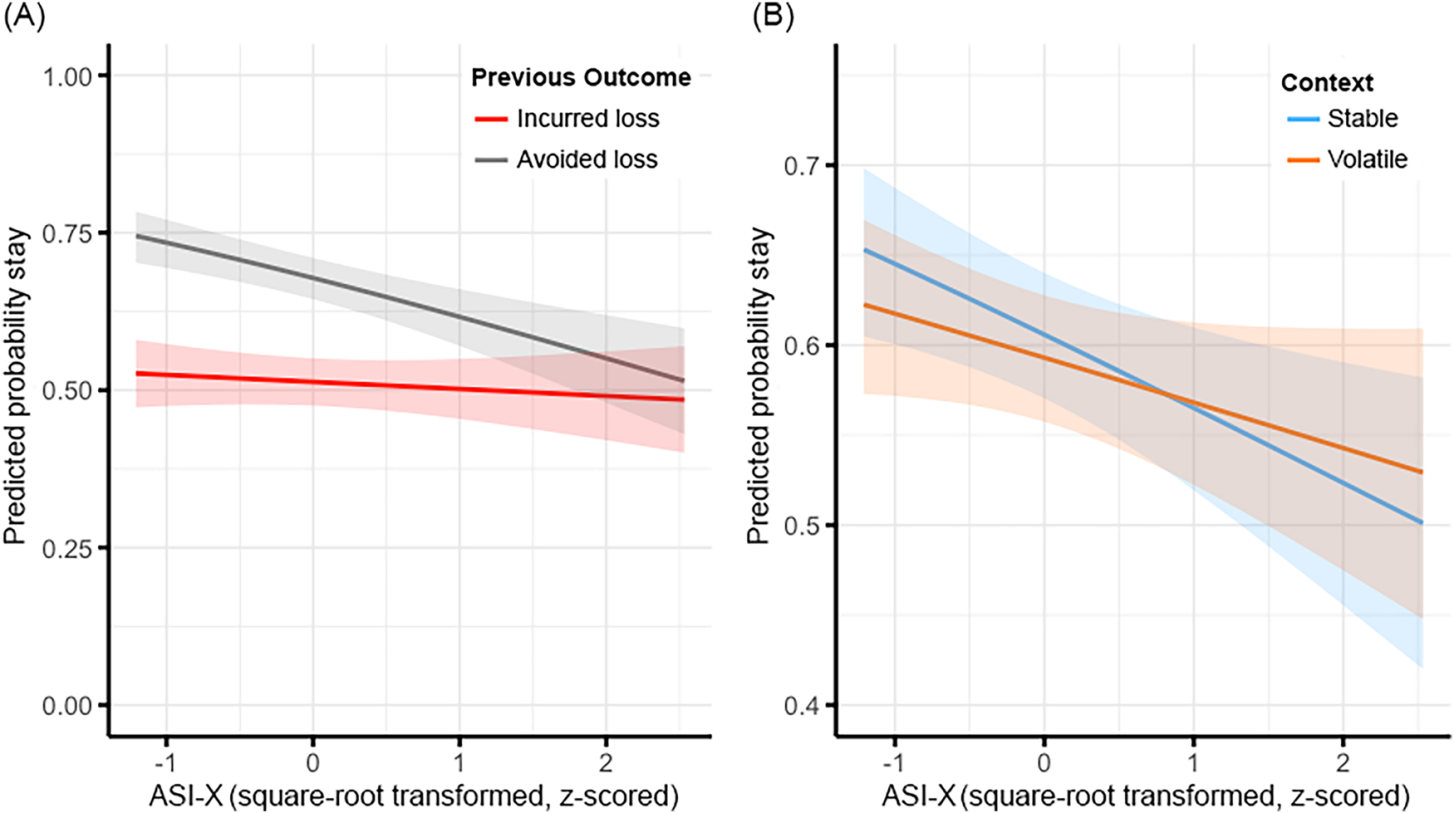
Cost Considerations Interact with Regular Substance Use to Predict Stay Choices. **A** Effect of ASI-X × previous outcome on stay choices. **B** Effect of ASI-X × context on stay choices. Shading around lines represents 95% confidence intervals for point estimates. ASI-X = Modified Addiction Severity Index.

Results from the individual differences regression (Eq. 2) indicated significant effects of ASI-X × previous outcome and ASI-X × context, but not ASI-X × previous outcome × context on stay choices (Table 1; Fig. 4). Simple slopes indicated that individuals with more years of regular use were less likely to stay on the same option after an avoided loss and less likely to stay on the same option in the stable context, contrary to hypotheses. Results were robust to measures of executive function (working memory, attention flexibility) and trait impulsivity (positive and negative urgency; see Table S6). These results suggest that, when comparing losses, individuals with more years of regular use less often adopted choice strategies (not repeating choices) that led to successful outcomes (avoided loss) and made more inconsistent choices in contexts where contingencies were stable. Supplemental Material includes additional tests probing the effects of ASI-X on choice outcomes (i.e., actual incurred loss amounts; Figure S9).

### Computational modeling

#### Model selection

The 2-level HGF with loss aversion parameter was the best-fitting model (Fig. 2b). Trial-by-trial simulated choices tracked observed choice behavior, validating our use of the model (Figure S5). See Supplemental Material for parameter distributions (Figure S10), trial-by-trial trajectories (Figure S11), and recovery (Table S8; Figures S12–S14).

#### Considering regular substance use

**Fig. 5:**
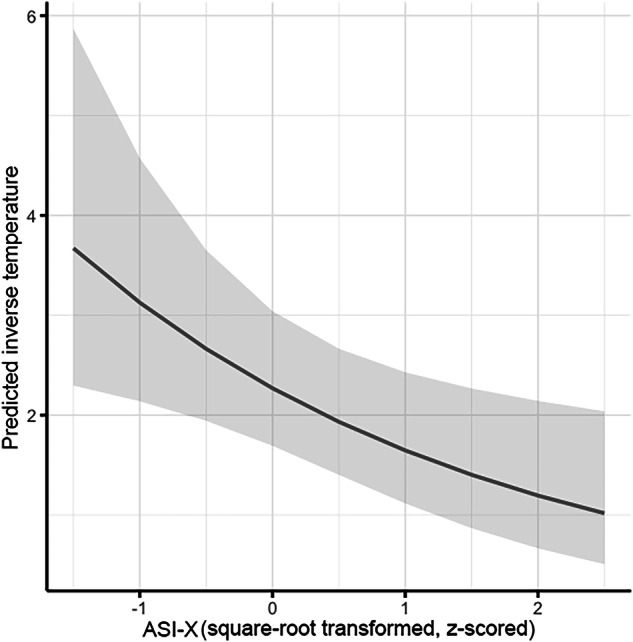
Regular Substance Use Predicts Choice Inconsistency. Lower values of inverse temperature parameter β reflect greater inconsistency in using expected values to guide choices. Shading around lines represents 95% confidence intervals for point estimates. ASI-X = Modified Addiction Severity Index.

Results from the HGF parameter regressions (Eq. 3) indicated a significant effect of ASI-X on inverse temperature, but not on volatility or loss aversion (Table 1; Fig. 5). Results held across various robustness checks (Table S7). These results suggest that those with more years of regular use less consistently used the expected values of their options to make choices.

## Discussion

A substantial body of theoretical [12, 13] and empirical research explores why individuals persist in substance use despite negative consequences, with particular emphasis on the role of decision-making processes in guiding such behavior. Experimental investigations have predominantly focused on the influence of rewards, single costs, or decision-making within singular contexts. Extending this work, the present study examines how regular substance use relates to the comparative evaluation of multiple costs across different learning environments. Following prior research [23, 33, 72, 73], individuals used cost outcomes to adjust their choices across stable and volatile contexts (particularly in the stable context), such that they more often adopted successful choice strategies (i.e., repeating choices after they avoided losses). However, individuals with more years of regular use tended not to use cost outcomes to adjust their choices across contexts, less often adopting successful choice strategies (i.e., *not* repeating choices after they avoided losses) regardless of whether contingencies were stable or changing. These individuals also showed more inconsistent choice behavior in the stable context, such that their choices appeared more random (i.e., not repeating choices overall) particularly when contingencies were stable. Finally, individuals with more years of regular use exhibited an underlying inconsistency using the difference in expected value between their options to guide choices across contexts (i.e., lower inverse temperature), such that the observed inconsistent choice behavior may have reflected difficulty consistently choosing the options with the best value. Overall, more years of regular use was associated with a pattern of decision-making that reflects an inconsistent use of cost information (whether past costs or expected values) to inform behavior.

The present study finds that, as expected, more variability in choice behavior occurred in the volatile context. We hypothesized that more years of regular use would be associated with perseveration (repeating choices) after an incurred loss in the volatile context. However, rather than seeing clear effects after loss and in the volatile condition, regular substance use was more strongly associated with not repeating choices after an *avoided* loss and not repeating choices overall in the *stable* context. For those with greater substance use severity, being consistent in choice behavior, particularly after something relatively positive happens, may be compromised due to decreased sensitivity to non-drug rewards (such as avoided monetary loss here, or instances when substance-related consequences were absent in the real world), which would make it difficult to recognize and continue behaviors that lead to effective outcomes [14, 74]. Further, inconsistency in choice behavior was most apparent in a context where costs were stable – and therefore most avoidable. It is possible that the stable context was more sensitive to inconsistent choice behavior because consistency in considering cost information was the only thing being assessed (vs. recognizing when to adjust or maintain choices in the volatile context). Additionally, theoretical work [16] suggests that those with greater substance use severity may assign too much importance to unexpected outcomes, treating a momentary loss or the absence of loss as a signal to switch rather than as noise, which could lead them to misinterpret fluctuations in otherwise stable environments as evidence that their current strategy is failing, prompting frequent switching despite stable contingencies. Altogether, these findings indicate that difficulty consistently using learned information to make appropriate choices is an important feature of substance use severity.

So, what might contribute to substance-related inconsistency in choice behavior? Our computational modeling results suggest that an underlying inconsistency in using expected values may contribute to inconsistent choice behavior. Years of regular substance use was associated with inconsistency using expected values to choose the “best” option, such that the impact of cost magnitudes and learned probabilities on choice behavior was blunted for individuals with more years of regular use. The blunted impact of magnitude and probabilities has been well-documented in studies testing decision-making using a variety of paradigms [20, 23, 72, 75, 76]. Additionally, researchers have suggested that executive functions (e.g., working memory [72], attention [23]), impulsivity [76], or affect may contribute to the blunted impact of expected values. One study testing the contribution of working memory to decision-making in a general sample found that poorer working memory was associated with more inconsistent choices [77], suggesting that differences in the ability to maintain and integrate information may impact effective decision-making (see also Supplemental Material). It is well-documented that impairments in executive function frequently co-occur with, can be a consequence of, and can be a risk factor for, substance use [78–80]. Given these findings, it may be important for future work to test the contribution of executive functions to consistency in the use of expected values to make choices. Additionally, theoretical models of addiction hypothesize that the immediate utility of substance use – frequently dependent on emotional state – overshadows its long-term costs, even if those costs are recognized [12, 13]. It is possible that emotional states such as distress could differentially impact the use of expected values to make choices (see also Supplemental Material). Future work could experimentally manipulate affect to better model how immediate states impact decision-making and choices.

Before concluding, we note limitations. First, loss magnitudes were fully randomized (past research uses magnitudes that are pseudo-randomized [47], in set order [50], fully randomized [48], or fixed [81]), which means that across participants, the frequency of a loss magnitude absolute difference of $1 or less between cards (e.g., -$1 vs. -$2) ranged from 69 to 101 of all trials, while the frequency of a loss magnitude absolute difference of $3 or greater between cards (e.g., -$1 vs. -$4) ranged from 6 to 25 of all trials. Given that loss sensitivity biases choices [82], experiencing more frequent magnitude differences of a greater size could impact choice behavior. However, the contribution of past outcomes to stay choices remained when controlling for differences in magnitude between the chosen and unchosen option, suggesting that consideration of outcomes remained consistent. Second, the probabilities used in each context were different (past research uses the same [47, 51] or different [46, 48, 50, 83] probability pairings in each context). A less-discriminable difference in probabilities in the stable context (i.e., 3-fold difference in the stable context vs. 4-fold difference in the volatile context) could contribute to inconsistent choices. However, trial-by-trial choice proportions (Figure S5) indicated that participants more often chose the card with the lower probability of loss across the stable context, suggesting that probabilities were discriminable. Third, choices involved monetary outcomes [84]. The use of monetary outcomes could raise questions about the contribution of socioeconomic status to costly choices (e.g., those lower in socioeconomic status could make costlier choices in the long run that provide immediate relief [85]) and the applicability of findings to choices involving actual substances. Correlations indicated that choices did not relate to socioeconomic status. Adapting reward-frame paradigms (that use monetary outcomes) to the loss domain is a logical first step in understanding cost comparisons. However, future work could test cost comparisons with more ecologically valid stimuli (e.g., testing choices when drug-related cues are present [86]). Fourth, our measure of substance use severity reflects only one indicator of severity: years of regular use. For example, we did not disaggregate current from past substance use. Current use, relative to past use, is associated with decision-making that is both riskier [87, 88] and comparable [18, 89, 90]. These mixed results suggest that future work can distinguish when and where differences between current and past use emerge in risky decision-making. More broadly, it will be important for future work to test how other indicators of substance use severity (e.g., abstinence stage, use amount, use-related problems) relate to cost comparisons.

Research on substance use and decision-making finds that people with greater substance use severity show heightened reward sensitivity, difficulty integrating the probabilities and magnitudes of their choice options, and insensitivity to costs. The present study suggests that the apparent insensitivity to costs may not reflect a lack of awareness or sensitivity, but rather an inconsistent use of cost information in guiding behavior. Clinically, individuals who use substances are not oblivious to consequences or incapable of learning from them. They recognize that their substance use has led to negative outcomes, that these outcomes feel aversive, and that they wish to avoid future consequences. However, when this knowledge is inconsistently used in decision-making, their behavior may appear perseverative, random, or irrational. Overall, the present study informs our conceptualization of substance use severity by highlighting that *inconsistency* in information use may serve as a key process driving continued substance use despite consequences.

## Supplementary information

Supplemental Material

## Data Availability

All data and code are available at https://osf.io/4e7nf/.
