# Supplementary material for: The relationship between regular substance use and cost comparisons in stable and volatile learning contexts
Source: Transl Psychiatry. 2026 Jan 30;16:103. doi: 10.1038/s41398-026-03830-z (PMC12923518; doi:10.1038/s41398-026-03830-z)
Supplement: Supplementary file 1 — Supplemental Material [file 41398_2026_3830_MOESM1_ESM.docx]

**Supplementary Methods**

**Instructions Given to Participants**

In this game, you will start with a $10 bonus. Your goal is to avoid losing money from your bonus by picking the correct box. You will choose between a WHITE box or a BLACK box. Inside each box, you'll see the amount of money you could lose. To choose the LEFT box, press the 'A' key on the keyboard. To choose the RIGHT box, press the 'L' key. Your choice will be highlighted in yellow. There will be many rounds in this game, and the box colors may change sides from round to round. Press RETURN key to continue.

In each round, there is a correct box and an incorrect box. If the box you choose is incorrect, you will lose the amount of money inside the box (between $1 and $5) from your $10 starting bonus. If the box is correct, you will not lose anything ($0). The correct box will be shown in the middle after you choose. The green bar at the bottom of the screen will show how much you have lost so far. Press RETURN key to continue.

For example, in this round you could lose either $5 or $2, depending on which box is correct. Let's say black is correct. If you chose black, you would not lose any money. But, if you chose white, you would lose $2 (the amount inside the white box) from your $10 bonus, and the green bar would get smaller. Press RETURN key to continue.

However, the chances that each color is correct are not equal. On any round, there might be a HIGHER or LOWER chance that one of the boxes is correct. By thinking about which color has been correct MORE OFTEN in the past, you can build a feeling for which color is MORE LIKELY to be correct in the current round. But, it is not always best to choose the color that is most likely to be correct. You should weigh up the CHANCE that the color is correct with the AMOUNT OF MONEY that you’ll lose if it turns out to be the wrong answer. Press RETURN key to continue.

At the end of the game, the money amounts from TWO of the rounds will be randomly chosen and subtracted from your starting $10 bonus. So, play every round as if it COULD count for your bonus. Any remaining money will be added onto your hourly pay for today's visit. You will always be paid $15 per hour -- your performance on this task determines how much MORE you can get (anywhere from $0 to $10 more). Remember, the green bar at the bottom of the screen tracks how much you lost so far. Press RETURN key to continue.

Please wait for the research assistant to explain this round! Then, you can try an example round. Press 'A' to choose the box on the left, or 'L' to choose the box on the right. The correct answer will be shown after you choose, and the green bar will keep track of how much you lost so far.

When you are ready, press the RETURN key to start the PRACTICE rounds!

Practice trials done! When you are ready, press the RETURN key to play the real game. Good luck!

**Measures to aid Interpretation of Task Validity**

***Executive Functions***

**Digit Span Test.** Research consistently finds poorer working memory and poorer cost-benefit decision-making among those with greater substance use severity ^1,2^. For example, one study found that individuals with a substance use disorder make costlier choices (choosing greater immediate gains in spite of greater future losses) and show poorer working memory than healthy comparisons. Among individuals who have a substance use disorder, those with the poorest working memory show the costliest decision-making ^3^. Another study found that individuals with a substance use disorder and those with poorer working memory were more sensitive to recent outcomes, as opposed to historical ones, in a probabilistic learning task ^4^. Altogether, research broadly suggests that working memory may constrain people’s ability to maintain and use cost information important for decision-making.

Working memory was estimated through the Digits Backward task from the Wechsler Digit Span Test ^5^, a component of the Wechsler Adult Intelligence Scale. Participants hear a series of numbers at a rate of one number per second, then are asked to repeat the numbers in reverse order. The number of digits increases by one until participants fail two consecutive series of the same length. Digits Backward measures participants’ ability to maintain and manipulate information held in working memory. Correct answers are summed to determine scores, where higher scores reflect greater working memory capacity.

**Trail Making Test (TMT).** Research frequently relates different features of attention such as flexibility ^6^ and sustained attention ^7^ to substance use and decision-making. For example, one study found that individuals with a substance use disorder show poorer flexibility in attention than healthy comparisons ^6^, and that those with poorer flexibility in attention (as well as a substance use disorder) make costlier choices (choosing greater immediate gains despite greater future losses). Attention allocation varies based on rewards, valuation, and motivations ^8,9^, which may in turn constrain individuals’ ability to track, use, and develop expectations about cost information.

Attention was estimated through the Trail Making Test-B ^10,11^. TMT-B requires participants to draw lines sequentially connecting 25 circled characters as quickly as possible, alternating between numbers and letters (e.g., 1, A, 2, B, 3, C) in three minutes. Time to completion is recorded in seconds, where higher scores reflect lower flexibility in attention. Three participants did not have complete TMT-B data, so were excluded from TMT-B analyses.

***Impulsivity***

**(Negative) Urgency, (lack of) Perseverance, (lack of) Premeditation, Sensation-Seeking, and Positive Urgency Scale (UPPS-P).** Research links dimensions of trait impulsivity to greater substance use severity and poorer decision-making ^12,13^. Negative and positive urgency are more robustly related to risky behaviors than other dimensions of trait impulsivity ^14-16^, and relate to use-related problems ^17^.

Impulsivity was estimated through the UPPS-P ^18^, which includes the subscales Negative Urgency (tendency to act rashly when experiencing negative affect), and Positive Urgency (tendency to act rashly when experiencing positive affect). For each item, participants indicate whether they Agree Strongly (1), Agree Some (2), Disagree Some (3), or Disagree Strongly (4). Responses for each subscale are summed, such that higher scores reflect greater negative and positive urgency.

***Personality Traits***

**Multidimensional Personality Questionnaire – Brief (MPQ-B).** The MPQ-B is a measure of personality that includes primary traits and broader dimensions of personality (e.g., negative emotionality) ^19^. Participants respond True (1 point) or False (0 points) to each statement. Responses are averaged, such that a higher score reflects stronger identification with the given trait.

To test discriminant validity of our loss adaptation of the probabilistic learning task, we selected the following primary trait scales: Achievement (drive for success), Absorption (propensity for imaginative and self-involving experiences), Social Potency (tendency to be decisive, influential, enjoy leadership). To replicate previous work relating anxiety/stress to learning on reward versions of the probabilistic learning task, we selected the Stress Reaction trait scale (tendency to be nervous, sensitive, and worried).

**Computational Modeling: HGF update equations**

In the 2-level HGF, the first level x_1_ at trial *t* corresponds to outcome u^t^ such that $x_{1}^{t}\in\left\{ 0, 1 \right\}$. Importantly, outcomes are coded in contingency space because the probabilities of loss associated with each card sum to 1. Therefore, outcomes are coded with respect to card 1, as:

$$u=\left\{ \begin{aligned} 0 if chose card 1 and incurred loss or if chose card 2 and avoided loss \\ 1 if chose card 2 and incurred loss or if chose card 1 and avoided loss \end{aligned} \right.$$

In other words, outcome *u* = 0 if card 1 would have/did incur loss, and *u* = 1 if card 2 would have/did incur loss. The estimated likelihood of either outcome occurring on trial *t* is a logistic sigmoid function of second-level belief $x_{2}^{t}$:

$$p\left( x_{1}^{t}=card 1 would have/did incur loss \right)\sim Bernoulli(s\left( x_{2}^{t} \right))$$

$$p\left( x_{1}^{t}= card 2 would have/did incur loss \right)\sim Bernoulli(1-s\left( x_{2}^{t} \right))$$

$$s\left( x_{2}^{t} \right)≝ \frac{1}{1+exp({-x}_{2}^{t})}$$

Second-level beliefs $x_{2}^{t}$ are drawn from a Gaussian random walk determined by the previous value $x_{2}^{t-1}$, with their step size (i.e., variance) parameterized by the volatility *ω*, as:

$$x_{2}^{t}\mathcal{\sim N(}x_{2}^{t-1},\exp\left( \omega\right))$$

Higher values of *ω* reflect more unstable choice-outcome associations, such that second-level beliefs are updated more rapidly. Values of *x* are updated trial-by-trial given new observed outcomes *u*:

$$x|u \sim\mathcal{N(}\mu_{x|u}, \pi_{x|u}^{-1})$$

For this equation, trial-by-trial *μ* values depend on outcomes *u*:

$\mu_{1}^{t}=\left\{ \begin{aligned} 0 if u=card 1 would have/did incur loss \\ 1 if u= card 2 would have/did incur loss \end{aligned} \right.$

$\mu_{2}^{t}= \mu_{2}^{t-1}+\varepsilon^{t}$, such that higher values of μ_2_ reflect a stronger belief that losses will be avoided when choosing card 1.

$\varepsilon^{t}=\frac{1}{\pi_{2}^{t}}\delta^{t}$, where second-level prediction error ε^t^ is the first-level prediction error δ^t^ weighted by the precision estimate $\frac{1}{\pi_{2}^{t}}$. The associated precision:

$\pi_{2}^{t}=\hat{\pi}_{2}^{t}+\frac{1}{\hat{\pi}_{1}^{t}}$ draws from the following estimates:

$\hat{\mu}_{1}^{t}≝s(\mu_{2}^{t-1})$, where first-level prediction $\hat{\mu}_{1}^{t}$ is a logistic sigmoid function of the previous trial’s mean second-level belief $\mu_{2}^{t-1}$

$\hat{\delta}^{t}≝\mu_{1}^{t}-\hat{\mu}_{1}^{t}$, where first-level prediction error δ^t^ is the difference between current belief $\mu_{1}^{t}$ and prediction $\hat{\mu}_{1}^{t}$

$\hat{\pi}_{1}^{t}≝ \frac{1}{\hat{\mu}_{1}^{t}(1-\hat{\mu}_{1}^{t})}$, where first-level precision $\pi_{1}^{t}$ is defined by mean beliefs $\mu_{1}^{t}$

$\hat{\pi}_{2}^{t}≝ \frac{1}{\sigma+e^{\omega}}$, where second-level precision $\pi_{2}^{t}$ is defined by the uncertainty σ (i.e., variance).

Finally, predictions are converted to action probabilities via the SoftMax response model:

$$p(choose card 2)= \frac{1}{1+exp(-\beta\times\left( q_{1}-q_{0} \right)\times\left( 2y-1 \right))}$$

$$y= \left\{ \begin{aligned} 0 if chose card 1 \\ 1 if chose card 2 \end{aligned} \right.$$

$$q_{1}={sign(loss magnitude}_{2})\times{{|loss matnitude}_{2}|}^{\rho}\times x$$

$$q_{0}={sign(loss magnitude}_{1})\times{{|loss matnitude}_{1}|}^{\rho}\times(1-x)$$

Here, x reflects the estimated probability that card 1 avoids loss (and that card 2 incurs loss) and parameter ρ estimates loss aversion (where greater values reflect greater loss aversion). Estimated probabilities are weighted by loss magnitudes of each option to compute expected values q_1_ and q_0_. The inverse temperature β reflects choice consistency (where higher values reflect more consistently choosing the option with the best expected value). See previous methodological papers for full details ^20,21^.

**Supplementary Results and Discussion**

**Validity of the Probabilistic Learning Task**

Distributions of measures included to aid interpretation of task validity are shown in Figure S3.

***Task Checks***

To determine how long it took participants to notice the context had changed and whether recognition varied by randomization, a repeated-measures ANOVAs tested for the effect of randomization and context on the number of trials and incurred losses before the first shift choice.

There was no significant main effect of randomization *F*(1, 135) = 0.478, *p* = .490), context, *F*(1, 135) = 0.159691, *p* = .806), or the interaction between context and randomization, *F*(1, 135) = 2.693, *p* = .103) on the number of *trials* before a shift. However, there was a significant interaction between context and randomization, *F*(1, 135) = 8.299, *p* = .005), but no significant main effects of randomization, *F*(1, 135) = 0.061, *p* = .806), or context, *F*(1, 135) = 0.971, *p* = .326) on the number of *losses* incurred before a shift. Simple effects analyses indicated that the number of incurred losses before the first shift choice differed between contexts when the stable context was first (*t*(135) = -2.786, *p* = .006), but not when the volatile context was first (*t*(135) = 1.229, *p* = .221). These results suggest that participants who experienced the stable context first may have been more willing to incur losses when the contexts changed because of the expectation that losses did not necessarily signal change in contingencies. Conversely, participants who experienced the volatile context first may have had a lower threshold for incurred losses before shifting, because losses more strongly signaled a contingency change in the volatile context (Figure S2). Given the effect of randomization on the loss outcome, we included randomization as a covariate.

Paired t-tests compared differences in summary metrics of task performance across task contexts: choice proportion expected value, lower magnitude loss, left option (indexing random choices), stayed on the same chosen option, stayed on the same chosen option after avoided loss, and stayed on the same chosen option after incurred loss; trial proportion of incurred losses; total summed loss; and mean reaction time.

A significant difference across task contexts was observed for: choice proportion best expected value (*t*(136) = 6.831, *p* = 10^-10^), trial proportion of incurred losses (*t*(136) = -4.314, *p* = 10^-5^) and total summed loss (*t*(136) = -3.658, *p* = .0003). No significant differences across task contexts were observed for: choice proportion lowest magnitude loss card (*t*(136) = -0.984, *p* = .327), choice proportion stayed (*t*(136) = 1.638, *p* = .104), choice proportion stayed after avoided loss (*t*(136) = 1.924, *p* = .056), choice proportion stayed after incurred loss (*t*(136) = -0.133, *p* = .894), choice proportion left option (*t*(136) = 1.492, *p* = .138), or mean reaction time (*t*(136) = -0.810, *p* = .420). These results suggest that participants lost more often chose the lower expected value option in the volatile context, thereby incurring more (and greater) losses (Figure S4).

***Replicating Basic Task Effects***

We additionally tested basic task effects following the approaches used with reward versions of the same task ^22^ to replicate established results. Two-sided Wilcoxon signed rank tests assessed basic task performance (i.e., choice proportion best expected value; choice proportion lower probability of loss; choice proportion lower magnitude loss) and choice proportion stayed after avoided loss compared to after incurred loss across task contexts.

Consistent with previous work, participants chose the option with the higher expected value (i.e. choice accuracy) more often than chance in the stable context (62.43 ± 1.23% (mean ± SEM), z = 8.26, p < 10^−16^) and in the volatile context (54.89 ± 0.94%, z = 4.06, p < 10^−5^). Participants chose the lower probability (of losing) option more often than chance in the stable context (62.99 ± 1.32%, mean ± SEM, z = 8.06, p < 10^−16^) and in the volatile context (54.84 ± 0.96%, z = 3.96, p < 10^−5^).

To ensure that participants integrated loss magnitudes with probability, we considered only trials where the option with the higher probability of losing had the “better” (i.e., lower) expected value of loss. Participants’ choices of the higher probability option did not differ from chance (stable: 44.88 ± 3.02%, z = -1.67, p = 0.953; volatile: 52.54 ± 5.28%, z = 0.47, p < 0.321). However, we noted that most participants had less than 4 (stable) or 1 (volatile) trials of this nature, out of 100 trials per block. In the stable context, participants stayed less on the high probability of loss option after they avoided loss than after they incurred loss (difference in choice proportion: -29.25± 2.95%, mean ± SEM, z = -7.46, p = 10^-14^), but stayed more on the low probability of loss option after they avoided loss than after they incurred loss (difference in choice proportion: 45.26 ± 1.46%, z = 10.1, p = 10^-16^). Results were similar for the volatile context. Here, participants also stayed less on the high probability of loss option after they avoided loss than after they incurred loss (difference in choice proportion: -37.80 ± 2.40%, z = -9.20, p < 10^−16^), but stayed more on the low probability of loss option after they avoided loss than after they incurred loss (difference in choice proportion: 58.79 ± 1.11%, z = 10.2, p < 10^−16^). However, participants stayed on the high probability of loss option after they avoided loss compared to after they incurred loss less in volatile than in stable contexts (difference volatile – stable: -9.26 ± 2.57%, z = -3.38, p = .0007). Consistent with previous work, they stayed on the low probability of loss option after they avoided loss compared to after they incurred loss more in volatile than stable contexts (difference volatile – stable: 13.53 ± 1.65%, z = 6.98, p < 10^−12^).

Overall, these results indicate that participants integrated loss magnitudes with probabilities in their choices. They suggest that participants used loss outcomes to inform future choices of the “better” (i.e., lower probability of loss) option across contexts, but particularly in the volatile context, consistent with prior research in the reward domain ^22-24^. These results align with established findings that participants are more sensitive to outcomes in the volatile compared to stable context, whether that be measured through model-free (e.g., win-stay/lose-shift) analyses or computational models ^25-27^. Figure S5 depicts trial-by-trial choices that also align with reward versions of the same task ^22,24^.

***Replicating Anxiety/Stress Effects***

To replicate prior studies testing the relationship between anxiety/stress, learning, and context ^26,28^, logistic regressions tested for relationships between MPQB stress reactivity and stay choices considering the previous outcome and task context (Table S4; table notes include model equation). Consistent with prior studies, results indicated that stress reactivity had a significant effect on consideration of the previous outcome in predicting stay choices (people with greater stress reactivity were less likely to stay on the same option after incurring loss relative to after avoiding loss) and on the consideration of both context and previous outcome in predicting stay choices (people with greater stress reactivity were more likely to stay on the same option after incurring loss in the volatile context relative to the stable context). This pattern of results is consistent with computational modeling of similar probabilistic learning tasks, which report that those higher on anxiety are more resistant to updating in volatile relative to stable contexts ^26,28^.

***Concurrent and Discriminant Validity***

To assess concurrent and discriminant validity, we tested for: (1) relationships between measures theoretically related to substance use severity (impulsivity; executive functions) and measures unrelated to substance use severity (trait achievement, absorption, and social potency) in predicting outcomes (stay choices; inverse temperature parameter) via regression when controlling for Age, Randomization, and loss magnitude difference; and (2) robustness of ASI-X predicting stay choices or inverse temperature when controlling for theoretically related measures (impulsivity; executive functions). Figure S8 shows zero-order correlations between all measures and task behavior; Tables S4 and S5 show results for relationships between measures and outcomes; and Tables S6 and S7 show robustness results for ASI-X.

First, regressions testing for relationships between measures and stay choices (Table S4) indicated that working memory and attention flexibility had significant effects on consideration of the previous outcome in predicting stay choices in the same direction as ASI-X (i.e., people with poorer working memory and cognitive flexibility were more likely to stay on the same option after incurring loss. Positive urgency and negative urgency had nonsignificant effects in mixed directions; while their effects on context in predicting stay choices were in the same direction as ASI-X (i.e., people with greater positive and negative urgency were more likely to stay on the same option in the volatile relative to stable context), their effects on previous outcome in predicting stay choices were in the opposite direction as ASI-X (i.e., people with greater positive and negative urgency were less likely to stay on the same option after incurred loss). However, trait achievement, absorption, and social potency did not have effects on consideration of the previous outcome in predicting stay choices. Regressions testing for relationships between measures and the inverse temperature parameter (Table S5) indicated that working memory capacity and positive urgency had a significant effect on inverse temperature in the *same* direction as ASI-X (i.e., people with poorer working memory and greater positive urgency showed poorer choice consistency). However, trait achievement, absorption, and social potency did not have effects on inverse temperature.

Second, regressions assessing the robustness of the relationship between ASI-X and outcomes when controlling for theoretically related measures indicated that the relationship between ASI-X and stay choices (Table S6) and ASI-X and inverse temperature (Table S7) were robust to all measures.

Altogether, task-derived behavior showed concurrent relationships with measures related to substance use severity (most notably, working memory) and discriminant relationships with unrelated measures (trait achievement, absorption, and social potency). The relationship between ASI-X and task behavior was also robust to all executive function measures and impulsivity. These results support the validity of this adaptation of the probabilistic learning task.

**Parameter Recovery**

To assess model fit, 100 simulations were run per participant with parameter recovery performed for 20 of the simulations. Correlations were estimated between simulated and observed percentage of same card choices and trajectories (across all trials, stable block, and volatile block), as well as recovered and true parameters (volatility ω, inverse temperature β, and loss aversion ρ). Figure S5 shows model-predicted trial-by-trial choices overlaid on observed choices. Figures S12 and S13 depict distributions of and correlations between true and recovered parameters and observed and simulated choices. Figure S14 shows simulated and observed mean belief trajectories.

The means and standard deviations for correlations between simulated and observed choices as well as between simulated and estimated parameters are presented in Table S8. Results suggest excellent recovery for all three parameters.

**Contribution of Regular Substance Use to Task Performance**

To assess relationships between regular substance use and task performance beyond choice strategies, a linear regression tested for the effect of ASI-X (square root-transformed, z-scored) on the total incurred loss amounts summed across the task controlling for age (mean-centered) and randomization. Results indicated significant effects for ASI-X on total incurred loss (β = 15.455, *SE* = 4.359, 95% CI = [6.831, 24.079], *t* = 3.546, *p* = .001). These results suggest that for every standard deviation increase in ASI-X, the predicted total incurred loss increases about $15. For zero-order correlation between ASI-X and total incurred loss, see Figure S1. For distribution of total incurred loss by ASI-X, see Figure S9.

**Table S1.** Sample Characteristics

| **Characteristic (*N* = 137)** | **Count** |
| --- | --- |
| Sex |  |
| Male | 92 |
| Female | 45 |
| Race/Ethnicity |  |
| Black | 55 |
| White | 40 |
| Hispanic | 13 |
| Asian | 12 |
| Mixed | 12 |
| Other | 3 |
| American Indian/Alaska Native | 2 |
| Education |  |
| Junior high/Middle school | 1 |
| Partially completed high school | 15 |
| Graduated high school | 33 |
| Partially completed college | 31 |
| Graduated college | 39 |
| Graduate degree | 18 |
|  | **[Min, Max], Mean (SD)** |
| Age | [18, 65], 40.53 (13.57) |
| ASI-X | [0, 154], 26.20 (33.40) |

*Note*. Min = minimum; Max = maximum; SD = standard deviation. ASI-X = Modified Addiction Severity Index.

**Table S2.** Means and Variances of HGF Priors

| **Model** | **Prior** | **Mean** | **Variance** | **Reference** |
| --- | --- | --- | --- | --- |
| **2-level HGF with loss aversion** | μ_2_^(0)^ | 0 | 0 | Default |
|  | μ_3_^(0)^ | 1 | 0 | Default |
|  | σ_2_^(0)^ | log(0.1) | 0 | Default |
|  | σ_3_^(0)^ | log(1) | 0 | Default |
|  | β | 0 | 1.5 | Follows log-normal distribution |
|  | ρ | 0 | 1 | Follows log-normal distribution with most probable value at 0.5, which follows square-root function |
|  | ω_2_ | -3 | 2 | Default |
|  | ω_3_ | 4 | 0 | Default |

*Note*. HGF = Hierarchical Gaussian Filter.

**Table S3.** Modified Addiction Severity Index Characteristics.

| **Pattern lifetime substance use endorsed** | **Count (*N* = 137)** | **% of total sample** |
| --- | --- | --- |
| No lifetime substance use | 4 | 3 |
| Any lifetime substance use | 133 | 97 |
| No regular use (using < 3 times/week) | 30 | 25 |
| Regular use (using 3+ times/week) | 103 | 75 |
| < 5 drinks of alcohol | 67 | 49 |
| 5+ drinks of alcohol | 43 | 31 |
| Heroin | 9 | 7 |
| Other opioids* | 9 | 7 |
| Methadone* | 2 | 1 |
| Cocaine/crack | 29 | 21 |
| Cannabis | 63 | 46 |
| Methamphetamine | 1 | <1 |
| Other amphetamines* | 3 | 2 |
| Hallucinogens | 7 | 5 |
| Inhalants | 3 | 2 |
| Nicotine | 73 | 53 |
| Other | 6 | 4 |
| Number of substances endorsed regular use |  |  |
| 1 | 24 | 18 |
| 2 | 18 | 13 |
| 3 | 24 | 18 |
| 4 | 18 | 13 |
| 5 | 11 | 8 |
| 6 | 3 | 2 |
| 7 | 3 | 2 |
| 8 | 1 | <1 |
| 9 | 1 | <1 |

*Note*. *Reflects use outside a prescription.

**Table S4.** Relationships Between Measures and Stay Choices

| **Measure** | **Term** | **β** | ***SE*** | **95% CI** | ***z*** | ***p*** |
| --- | --- | --- | --- | --- | --- | --- |
| ASI-X | × Context | 0.083 | 0.037 | 0.010, 0.155 | 2.243 | .025 |
|  | × Previous outcome | 0.226 | 0.038 | 0.152, 0.300 | 6.014 | 10^-9^ |
|  | × Context × Previous outcome | -0.038 | 0.053 | -0.141, 0.066 | -0.713 | .476 |
| MPQB Stress Reactivity | × Context | -0.013 | 0.037 | -0.085, 0.058 | -0.369 | .712 |
|  | × Previous outcome | -0.085 | 0.038 | -0.159, -0.012 | -2.269 | .023 |
|  | × Context × Previous outcome | 0.109 | 0.053 | 0.006, 0.213 | 2.068 | .039 |
| UPPS-P Negative Urgency | × Context | 0.013 | 0.036 | -0.058, 0.083 | 0.351 | .726 |
|  | × Previous outcome | -0.064 | 0.037 | -0.137, 0.009 | -1.727 | .084 |
|  | × Context × Previous outcome | 0.092 | 0.052 | -0.010, 0.195 | 1.766 | .077 |
| UPPS-P Positive Urgency | × Context | 0.001 | 0.036 | -0.069, 0.072 | 0.041 | .967 |
|  | × Previous outcome | -0.001 | 0.037 | -0.074, 0.072 | -0.023 | .981 |
|  | × Context × Previous outcome | 0.131 | 0.052 | 0.028, 0.234 | 2.494 | .013 |
| Digits Backward* | × Context | 0.067 | 0.038 | -0.007, 0.140 | 1.775 | .076 |
|  | × Previous outcome | 0.228 | 0.039 | 0.152, 0.04 | 5.901 | 10^-9^ |
|  | × Context × Previous outcome | -0.056 | 0.054 | -0.162, 0.050 | -1.036 | .300 |
| Trail-Making Test B | × Context | 0.084 | 0.037 | 0.012, 0.157 | 2.278 | .023 |
|  | × Previous outcome | 0.313 | 0.038 | 0.239, 0.387 | 8.310 | 10^-16^ |
|  | × Context × Previous outcome | -0.103 | 0.053 | -0.207, 0.001 | -1.948 | .051 |
| MPQB Achievement | × Context | -0.031 | 0.036 | -0.101, 0.040 | -0.854 | .393 |
|  | × Previous outcome | -0.042 | 0.037 | -0.115, 0.031 | -1.136 | .256 |
|  | × Context × Previous outcome | 0.077 | 0.052 | -0.025, 0.179 | 1.475 | .140 |
| MPQB Absorption | × Context | 0.002 | 0.036 | -0.068, 0.072 | 0.063 | .950 |
|  | × Previous outcome | 0.004 | 0.037 | -0.068, 0.076 | 0.105 | .916 |
|  | × Context × Previous outcome | 0.035 | 0.052 | -0.067, 0.136 | 0.671 | .502 |
| MPQB Social Potency | × Context | -0.049 | 0.037 | -0.121, 0.023 | -1.341 | .180 |
|  | × Previous outcome | 0.049 | 0.037 | -0.025, 0.122 | 1.301 | .193 |
|  | × Context × Previous outcome | 0.031 | 0.053 | -0.072, 0.134 | 0.585 | .559 |

*Note*. ASI-X = Modified Addiction Severity Index. MPQB = Multidimensional Personality Questionnaire Brief; UPPS-P = (Negative) Urgency, (lack of) Perseverance, (lack of) Premeditation, Sensation-Seeking, and Positive Urgency Scale. We used the binary logistic regression equation: $Stay \sim Context \times Previous outcome\times Measure+Loss magnitude difference +Age +Randomization+\left( 1 | Subject \right).$ Note that each measure in the first column was its own model, so the table reflects the results of 9 separate regressions. ASI-X results are provided for reference. *For ease of comparison, Digits Backward is reverse scored so that greater values reflect poorer working memory.

**Table S5.** Relationships Between Measures and Inverse Temperature

| **Term** | **β** | ***SE*** | **95% CI** | ***t*** | ***p*** |
| --- | --- | --- | --- | --- | --- |
| ASI-X | -0.321 | 0.013 | -0.570, -0.072 | -2.547 | .012 |
| MPQB Stress Reactivity | -0.016 | 0.113 | -0.238, 0.207 | -0.138 | .890 |
| UPPS-P Negative Urgency | -0.211 | 0.110 | -0.428, 0.006 | -1.923 | .057 |
| UPPS-P Positive Urgency | -0.249 | 0.109 | -0.465, -0.033 | -2.280 | .024 |
| Digits Backward* | -0.350 | 0.110 | -0.568, -0.133 | -3.183 | .002 |
| Trail-Making Test B | -0.206 | 0.120 | -0.443, 0.030 | -1.725 | .087 |
| MPQB Achievement | -0.013 | 0.111 | -0.233, 0.207 | -0.114 | .909 |
| MPQB Absorption | -0.176 | 0.114 | -0.401, 0.048 | -1.553 | .123 |
| MPQB Social Potency | -0.080 | 0.111 | -0.299, 0.140 | -0.715 | .476 |

*Note*. ASI-X = Modified Addiction Severity Index; MPQB = Multidimensional Personality Questionnaire Brief; UPPS-P = (Negative) Urgency, (lack of) Perseverance, (lack of) Premeditation, Sensation-Seeking, and Positive Urgency Scale. The following linear regression equation was used: $Inverse temperature \sim Measure+Age +Randomization$. Note that each measure in the first column was its own model, so the table reflects the results of 9 separate regressions. ASI-X results are provided for reference. *For ease of comparison, Digits Backward is reverse scored so that greater values reflect poorer working memory.

**Table S6.** Effect of ASI-X on Stay Choices when Controlling for Related Measures

| **Measure controlled for** | **Term** | **β** | ***SE*** | **95% CI** | ***z*** | ***p*** |
| --- | --- | --- | --- | --- | --- | --- |
| UPPS-P Negative Urgency | ASI-X × Context | 0.083 | 0.037 | 0.010, 0.155 | 2.243 | .025 |
|  | ASI-X × Previous outcome | 0.226 | 0.038 | 0.152, 0.300 | 6.015 | 10^-9^ |
|  | ASI-X × Context × Previous outcome | -0.038 | 0.053 | -0.141, 0.066 | -0.713 | .476 |
| UPPS-P Positive Urgency | ASI-X × Context | 0.083 | 0.037 | 0.010, 0.155 | 2.243 | .025 |
|  | ASI-X × Previous outcome | 0.226 | 0.038 | 0.152, 0.300 | 6.014 | 10^-9^ |
|  | ASI-X × Context × Previous outcome | -0.038 | 0.053 | -0.141, 0.066 | -0.712 | .476 |
| Digits Backward* | ASI-X × Context | 0.083 | 0.037 | 0.010, 0.155 | 2.243 | .025 |
|  | ASI-X × Previous outcome | 0.226 | 0.038 | 0.153, 0.230 | 6.016 | 10^-9^ |
|  | ASI-X × Context × Previous outcome | -0.038 | 0.053 | -0.141, 0.066 | -0.711 | .477 |
| Trail-Making Test B | ASI-X × Context | 0.074 | 0.037 | 0.0002, 0.147 | 1.966 | .049 |
|  | ASI-X × Previous outcome | 0.230 | 0.038 | 0.155, 0.305 | 6.010 | 10^-9^ |
|  | ASI-X × Context × Previous outcome | -0.020 | 0.054 | -0.125, 0.086 | -0.367 | .714 |

*Note*. ASI-X = Modified Addiction Severity Index; UPPS-P = (Negative) Urgency, (lack of) Perseverance, (lack of) Premeditation, Sensation-Seeking, and Positive Urgency Scale. The following binary logistic regression equation was used: $Stay \sim Context \times Previous outcome\times\text{ASI-X}+ Measure+Loss magnitude difference +Age +Randomization+\left( 1 | Subject \right)$. Note that each measure in the first column was a covariate in a separate model, so the table reflects the results of 4 separate regressions. *For ease of comparison, Digits Backward is reverse scored so that greater values reflect poorer working memory.

**Table S7.** Effect of ASI-X on Inverse Temperature when Controlling for Related Measures

| **Measure controlled for** | **β** | ***SE*** | **95% CI** | ***t*** | ***p*** |
| --- | --- | --- | --- | --- | --- |
| UPPS-P Negative Urgency | -0.278 | 0.132 | -0.540, -0.016 | -2.101 | .038 |
| UPPS-P Positive Urgency | -0.271 | 0.132 | -0.532, -0.011 | -2.059 | .042 |
| Digits Backward* | -0.253 | 0.126 | -0.502, -0.003 | -2.004 | .047 |
| Trail-Making Test B | -0.332 | 0.127 | -0.583, -0.081 | -2.615 | .010 |

*Note*. ASI-X = Modified Addiction Severity Index; MPQB = Multidimensional Personality Questionnaire Brief; UPPS-P = (Negative) Urgency, (lack of) Perseverance, (lack of) Premeditation, Sensation-Seeking, and Positive Urgency Scale. The following linear regression equation was used: $Inverse temperature \sim\text{ASI-X}+Measure+Age +Randomization$. The effects shown are for the ASI-X term when controlling for the listed measure in the first column. Note that each measure in the first column was a covariate in a separate model, so the table reflects the results of 4 separate regressions. *For ease of comparison, Digits Backward is reverse scored so that greater values reflect poorer working memory.

**Table S8.** Pearson Correlation Coefficients for Parameter Recovery

| **Task Context** | **Parameter** | **Mean *R*** | **SD** |
| --- | --- | --- | --- |
| **Stable** | Choice | 0.919 | 0.008 |
|  | μ_2_ | 0.834 | 0.079 |
|  | σ_2_ | 0.801 | 0.103 |
|  | ε_2_ | 0.818 | 0.131 |
| **Volatile** | Choice | 0.514 | 0.039 |
|  | μ_2_ | 0.644 | 0.205 |
|  | σ_2_ | 0.702 | 0.190 |
|  | ε_2_ | 0.727 | 0.208 |
| **All** | Choice | 0.859 | 0.012 |
|  | ω | 0.906 | 0.013 |
|  | β | 0.982 | 0.010 |
|  | λ | 0.751 | 0.091 |

*Note*. SD = Standard deviation.

**Figure S1.** Zero-Order Spearman Correlations across Individual Difference Measures and Task Parameters.


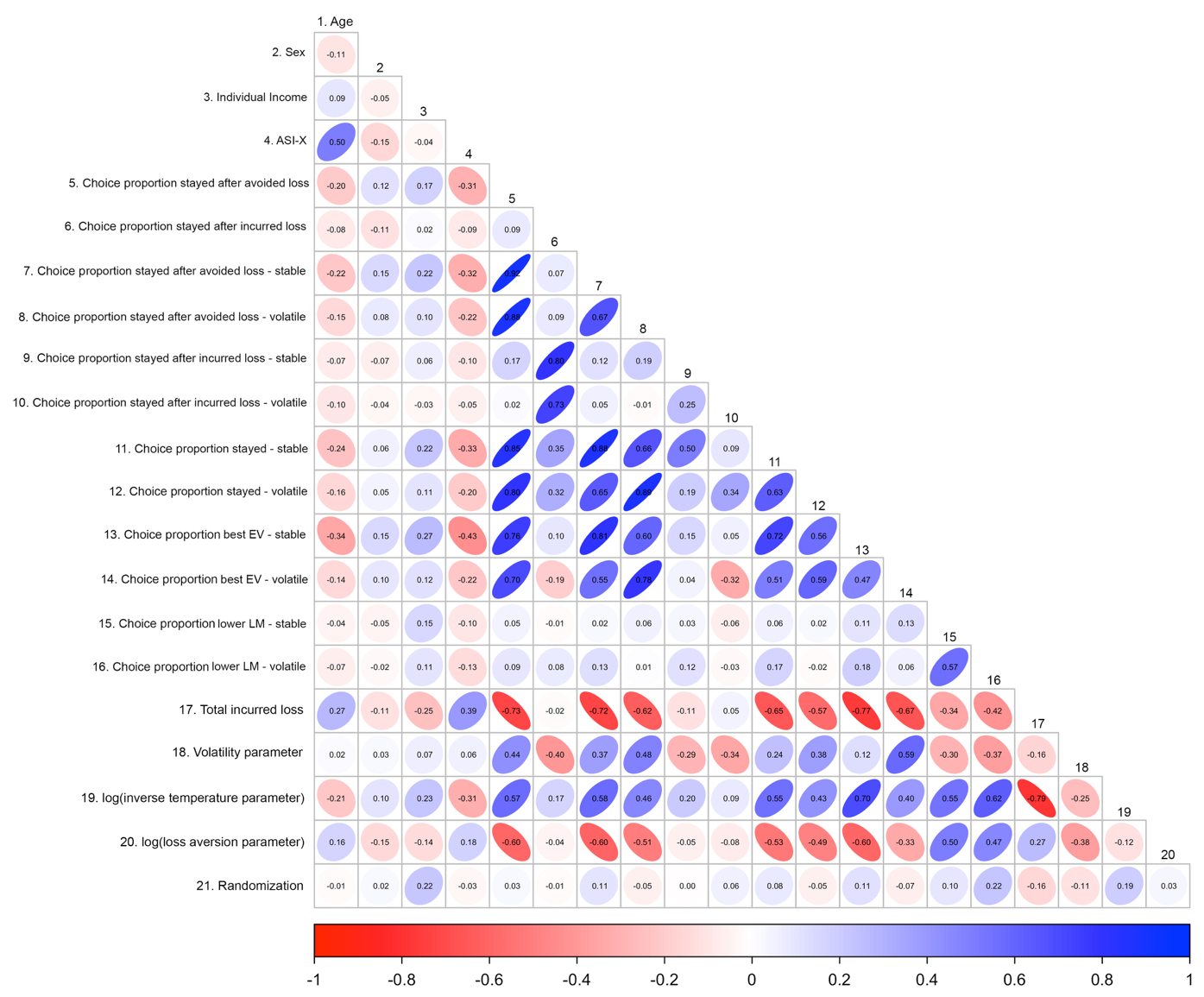


*Note*. ASI-X = Modified Addiction Severity Index; EV = expected value; LM = loss magnitude. Higher values of volatility parameter ω reflect faster belief updating, higher values of inverse temperature β reflect greater consistency using expected values to guide choices, and higher values of loss aversion ρ parameter reflect greater sensitivity to loss.

**Figure S2.** Distribution of Stay Choices Before First Shift Choice
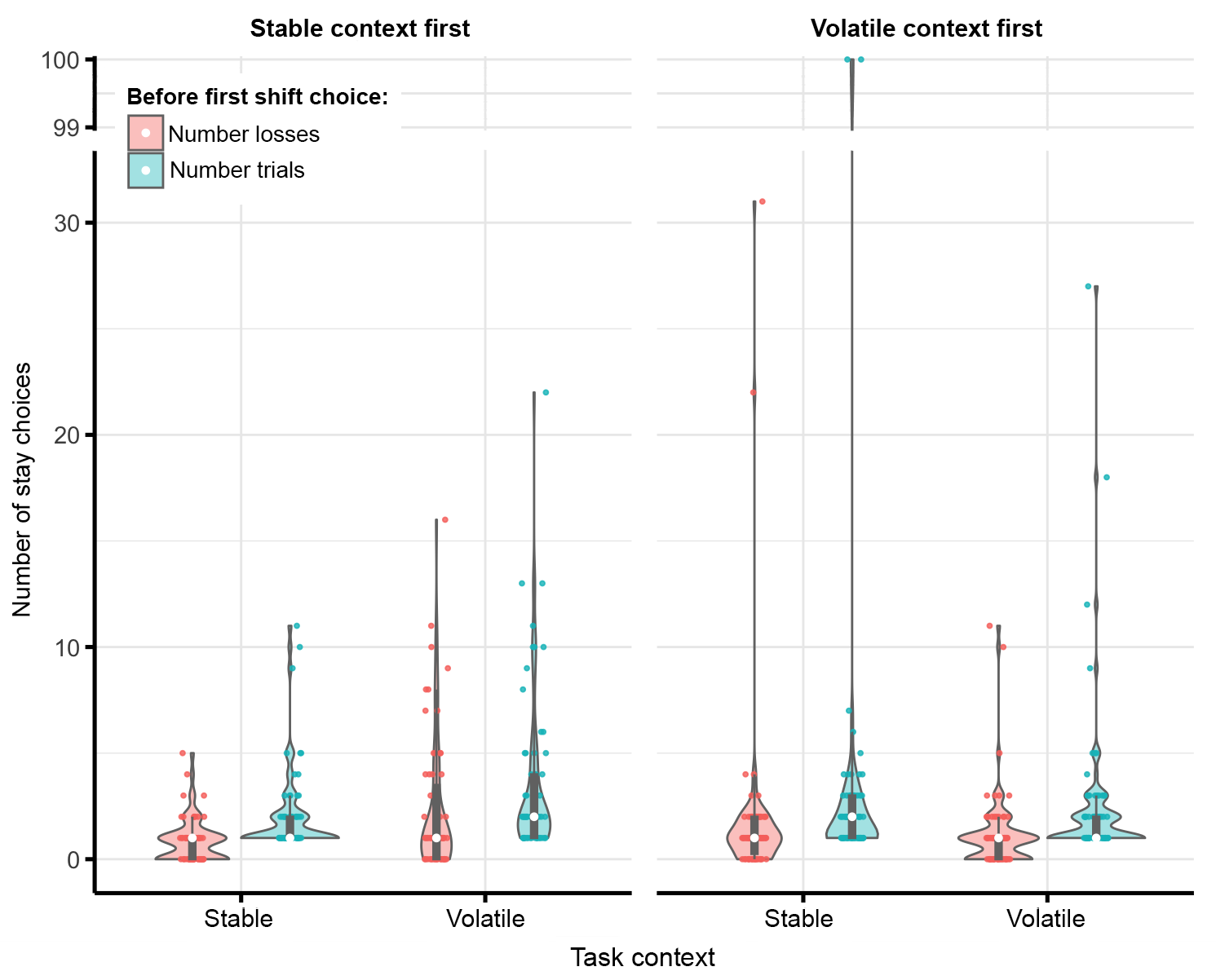


*Note*. For visualization purposes, y-axis break occurs at 32 to 99.

**Figure S3.** Distributions of Individual Difference Variables


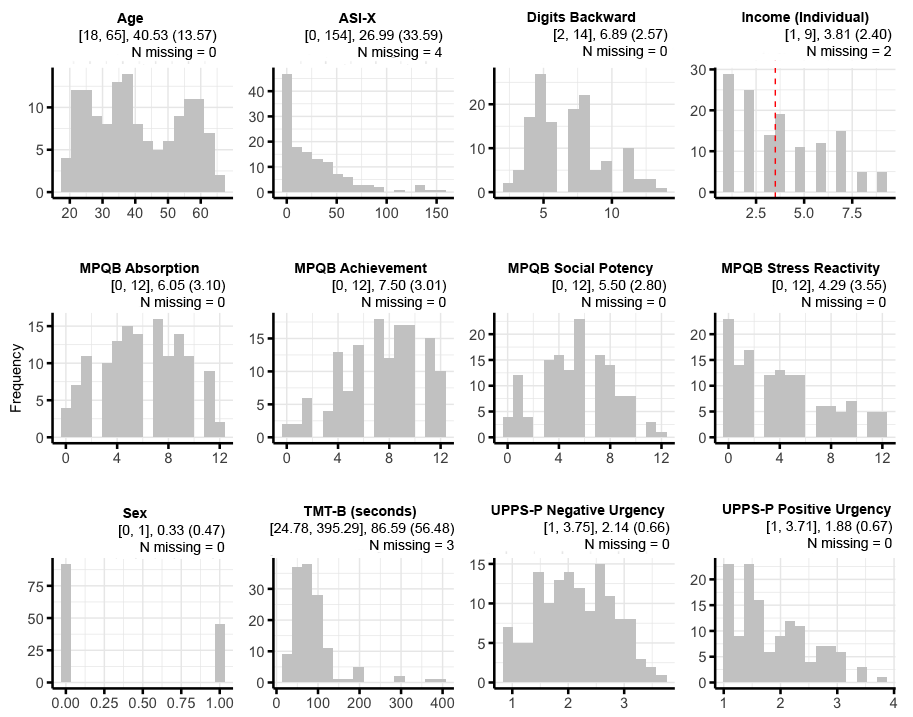


*Note*. [Minimum, maximum], mean (standard deviation). ASI-X = Modified Addiction Severity Index; MPQB = Multidimensional Personality Questionnaire Brief; TMT-B = Trail Making Test B; UPPS-P = (Negative) Urgency, (lack of) Perseverance, (lack of) Premeditation, Sensation-Seeking, and Positive Urgency Scale. Sex reflects sex assigned at birth, where: 0 = Male, 1 = Female. Income (Individual) reflects total individual income before taxes in the past 12 months, where: 1 = Less than $5,000, 2 = $5,000 through $11,999; 3 = $12,000 through $15,999; 4 = $16,000 through $24,999; 5 = $25,000 through $34,999; 6 = $35,000 through $49,999; 7 = $50,000 through $74,999; 8 = $75,000 through $99,999; 9 = $10,000 and greater. Dashed red line represents federal poverty line for an individual living in Connecticut ($15,650).

**Figure S4.** Distributions of Choice Proportions


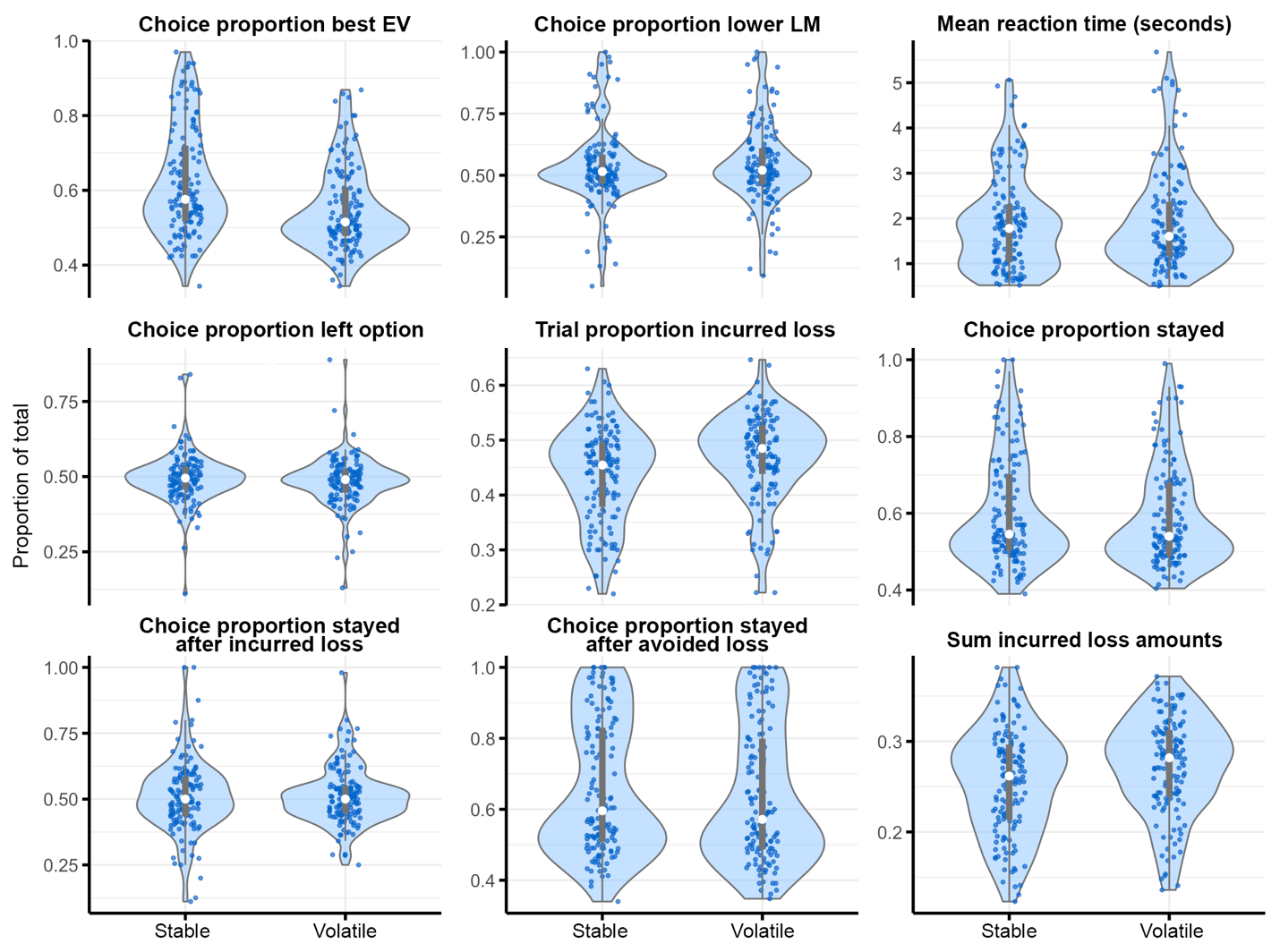


*Note*. EV = Expected Value; LM = Loss Magnitude. For visualization purposes, summed incurred loss amounts are divided by the total possible summed loss per task context.

**Figure S5.** Trial-by-Trial Choices


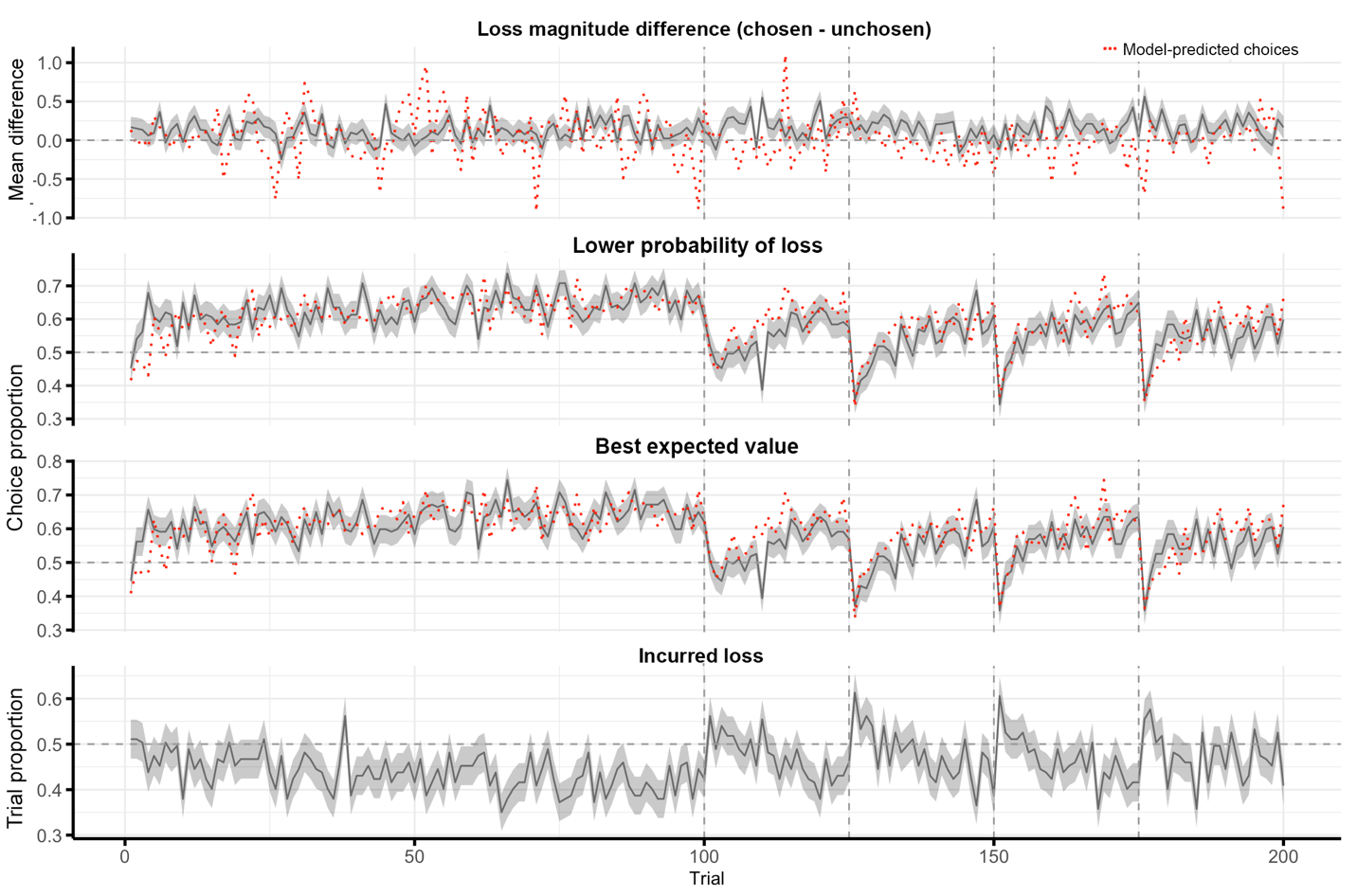


*Note*. For visualization purposes, trial numbers were adjusted so that the first 100 trials reflect choices from the stable context and the last 100 trials reflect choices from the volatile context. Dotted red line reflects choices predicted by 2-level HGF with loss aversion model. Across participants, the choice proportion of the best expected value was greater than chance across the stable context (indicating recognition that contingencies were stable) and sharply dropped then increased every 25 trials in the volatile context (indicating recognition that contingencies changed every 25 trials). The difference between loss magnitudes of the selected card and the unselected card was greater than zero across trials (indicating preference for smaller loss magnitudes) and did not fluctuate according to contingency changes, indicating that sensitivity to loss was consistent across the task.

**Figure S6.** Distributions of Incurred Losses and Loss Magnitudes by Side

**
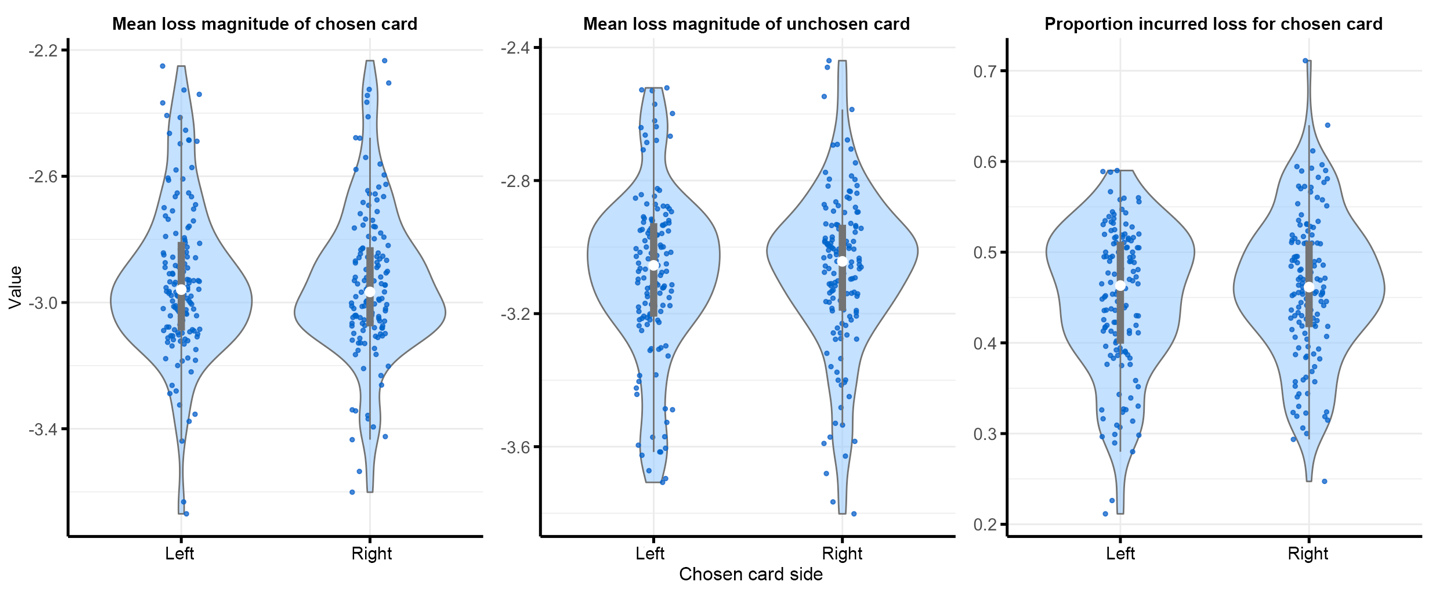
**

**Figure S7.** Distributions of Proportion Incurred Loss vs. True Probability of Loss


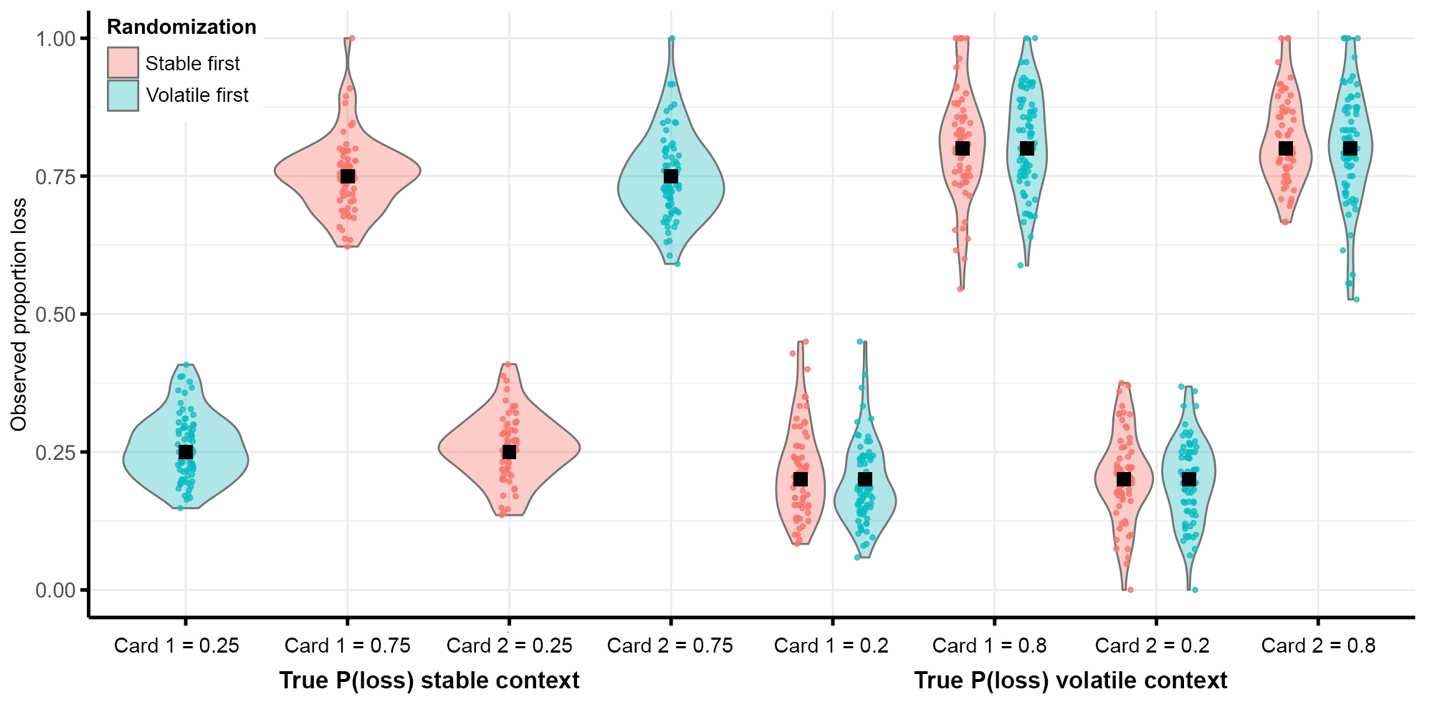


*Note*. Black squares represent true probability of loss for each card. Note that in the stable context, the probabilities of loss associated with each card do not switch. When the stable context is first, card 1’s probability of loss during stable is 0.75 only. When the volatile context is first, card 1’s probability of loss during stable is 0.25 only.

**Figure S8.** Zero-Order Spearman Correlations across Related Measures and Task Parameters.

**
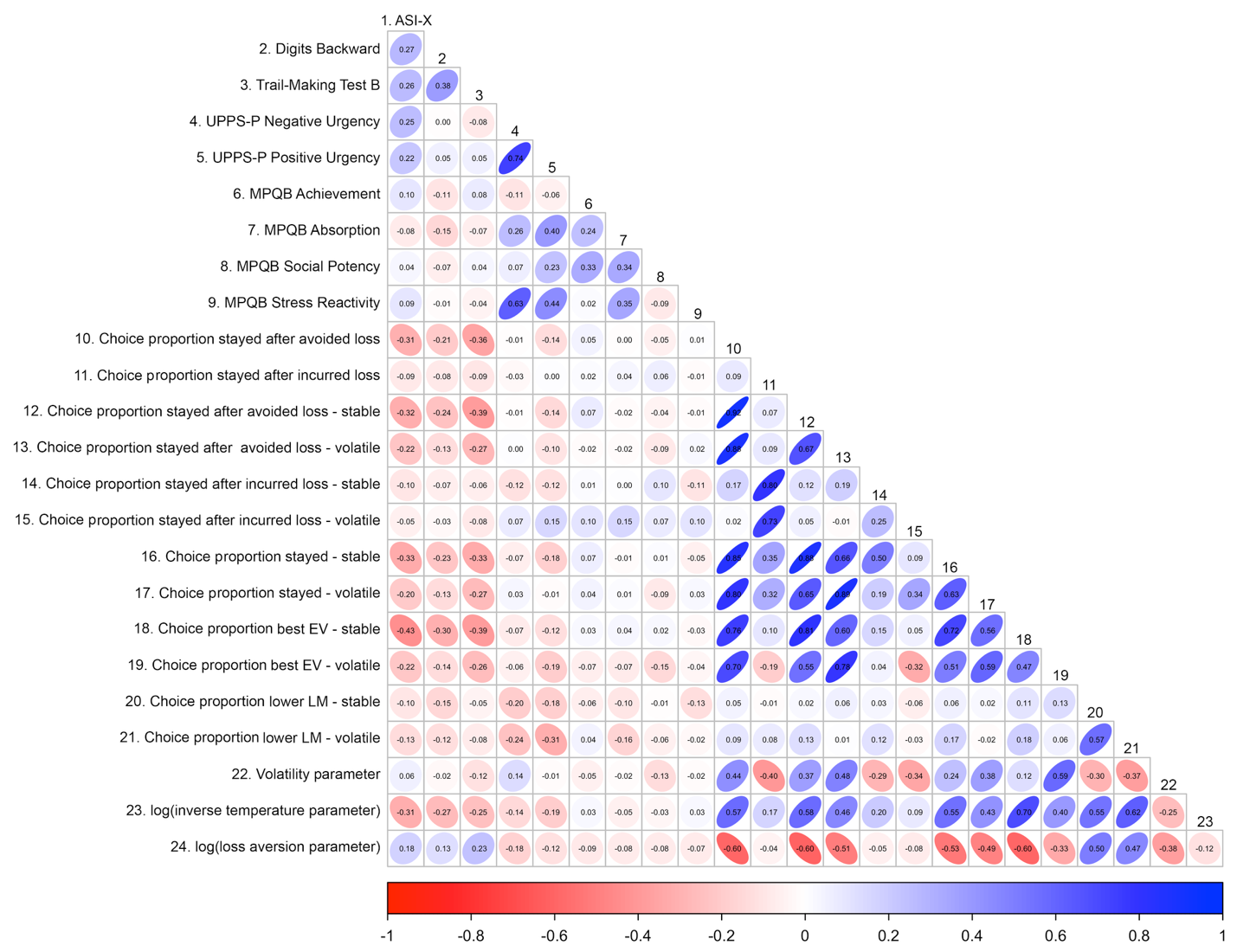
**

*Note.* ASI-X = Modified Addiction Severity Index; EV = expected value; LM = loss magnitude; MPQB = Multidimensional Personality Questionnaire Brief; UPPS-P = (Negative) Urgency, (lack of) Perseverance, (lack of) Premeditation, Sensation-Seeking, and Positive Urgency Scale. Higher values of volatility parameter ω reflect faster belief updating, higher values of inverse temperature β reflect greater consistency using expected values to guide choices, and higher values of loss aversion ρ parameter reflect greater sensitivity to loss.

**Figure S9.** Distribution of Total Incurred Loss


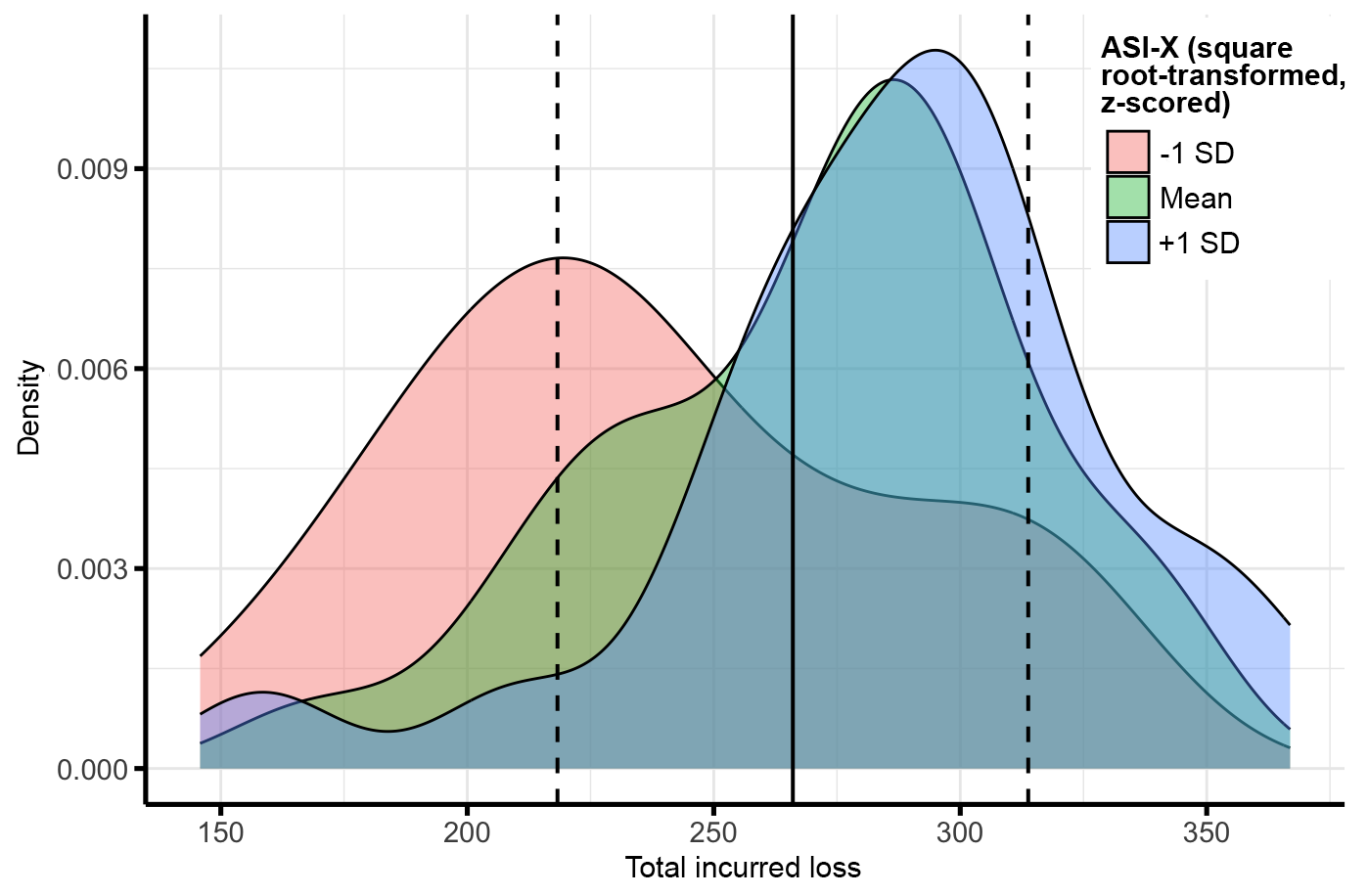


*Note*. ASI-X = Modified Addiction Severity Index. SD = Standard Deviation. Total incurred loss represents the total incurred loss amounts summed across the task. For visualization purposes, ASI-X was binned into sample mean and one standard deviation above and below the mean. The solid vertical line represents the mean total incurred loss of the sample, and the dashed vertical lines represent one standard deviation above and below the mean total incurred loss of the sample.

**Figure S10.** Distributions of 2-Level HGF with Loss Aversion Parameters


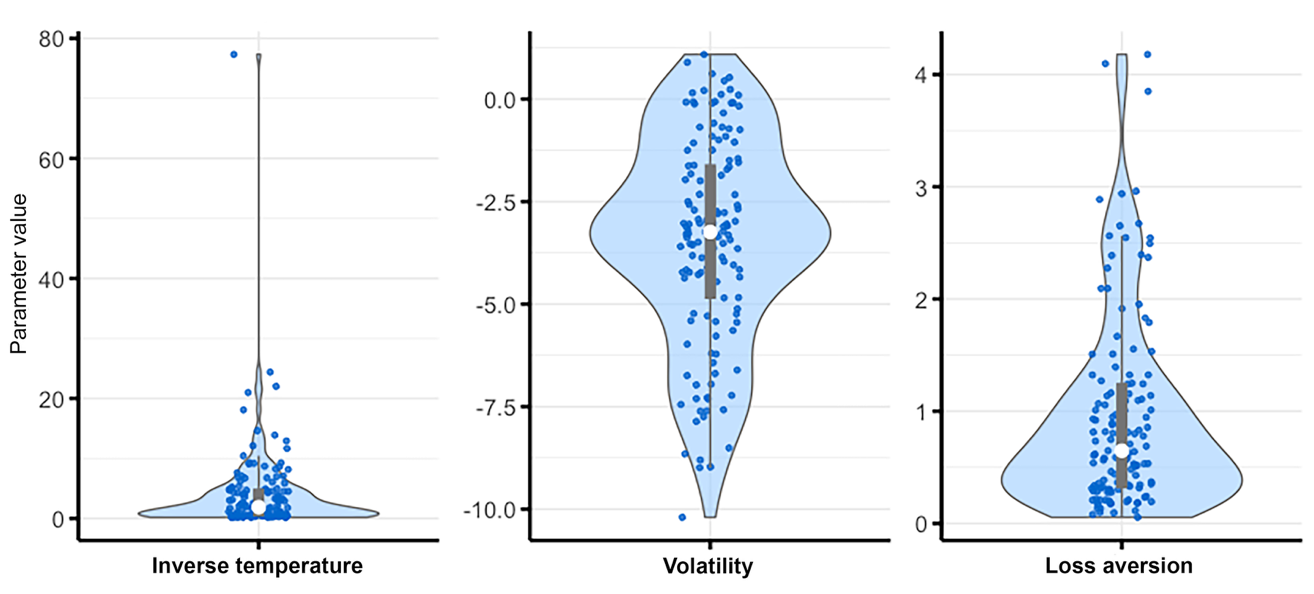


*Note.* Higher values of volatility ω reflect faster belief updating, higher values of inverse temperature β reflect greater consistency using expected values to guide choices, and higher values of loss aversion ρ parameter reflect greater sensitivity to loss.

**Figure S11.** 2-Level HGF with Loss Aversion Mean Belief Trajectories

**
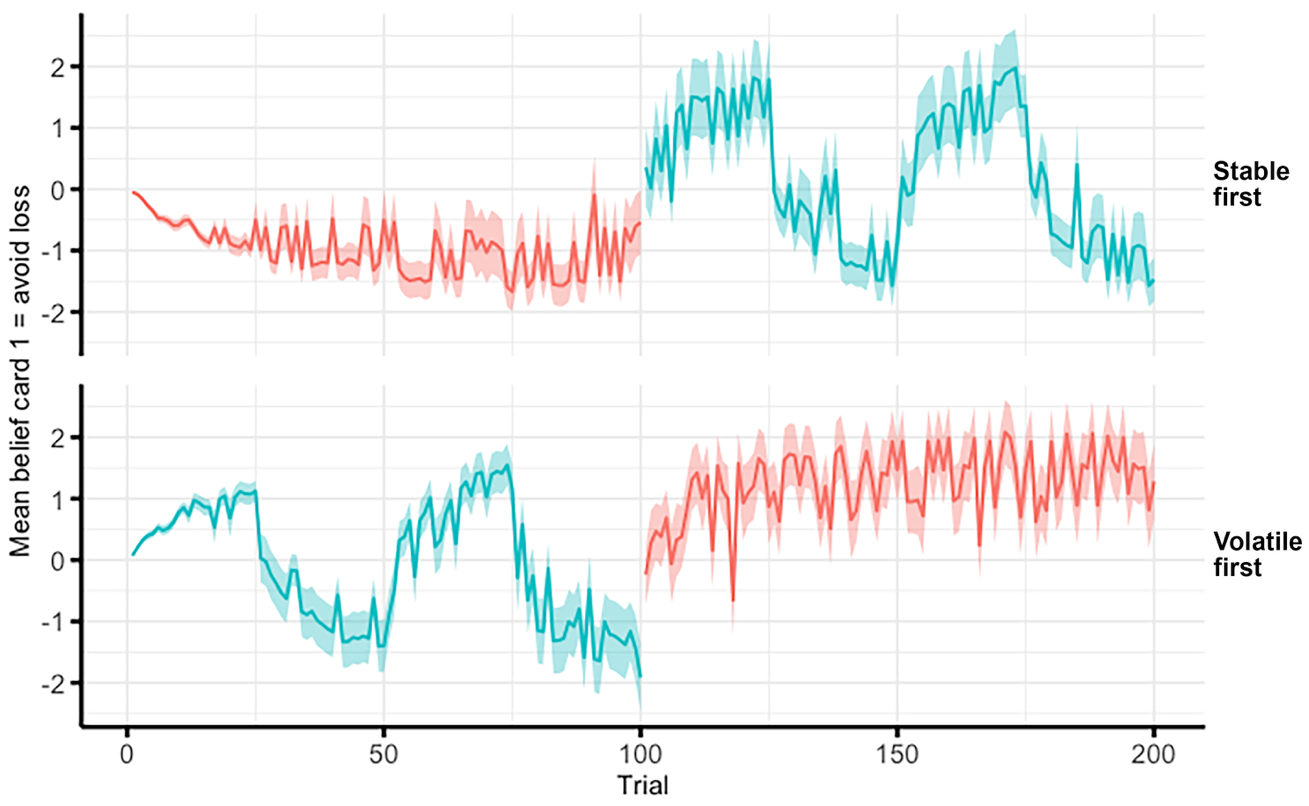
**

*Note*. True probabilities that card 1 = avoided loss for (1) Stable first randomization: 0.25 for 100 trials, 0.8 for 25 trials, 0.2 for 25 trials, 0.8 for 25 trials, 0.2 for 25 trials; (2) Volatile first randomization: 0.8 for 25 trials, 0.2 for 25 trials, 0.8 for 25 trials, 0.2 for 25 trials, 0.75 for 100 trials.

**Figure S12.** 2-Level HGF with Loss Aversion Recovery and Simulation


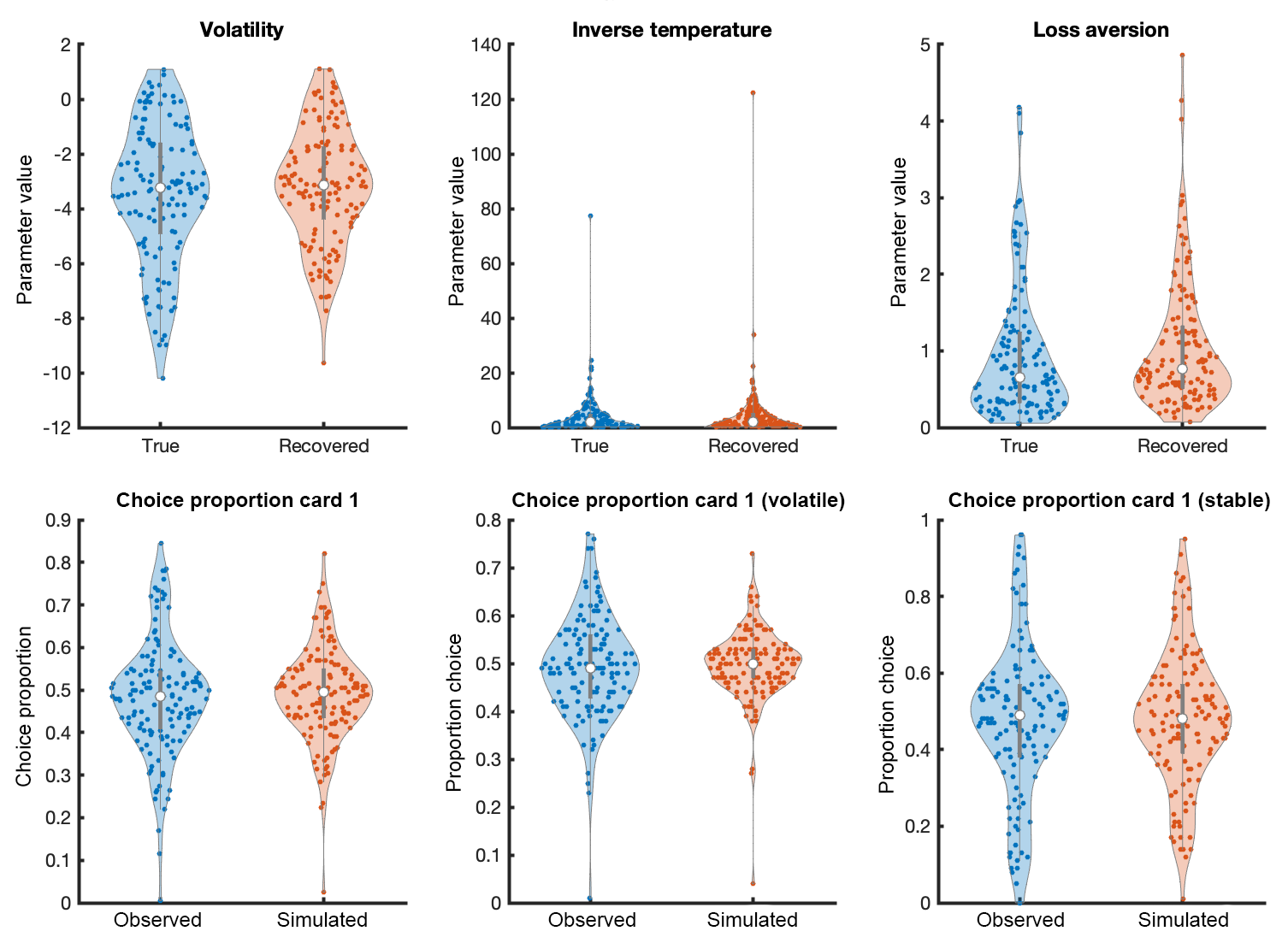


*Note*. True and recovered parameters (top panel); observed (blue) and simulated (orange) choice behavior (bottom panel).

**Figure S13.** 2-Level HGF with Loss Aversion Recovery and Simulation Correlations
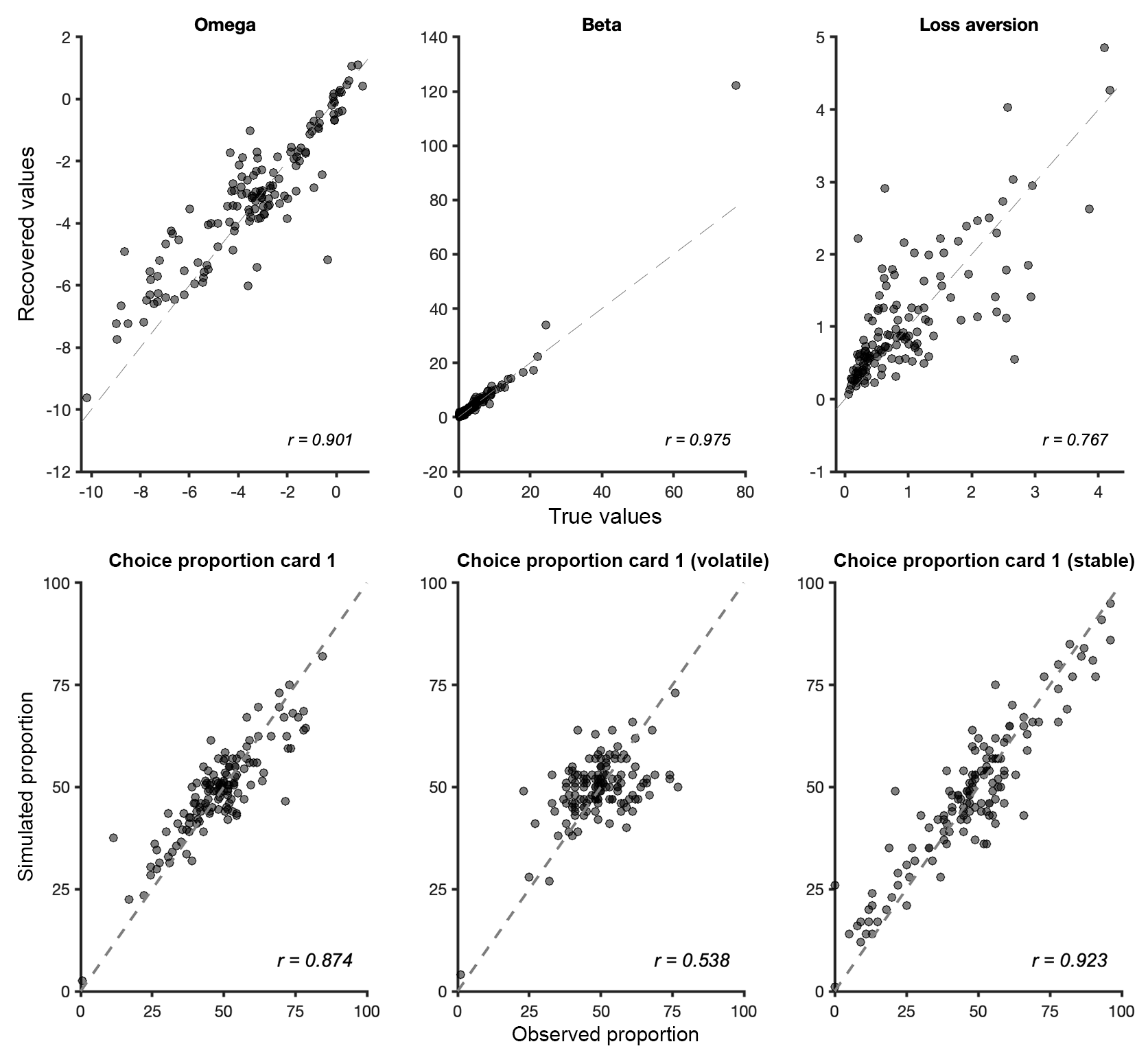


*Note*. Correlations shown for one of 20 simulations.

**Figure S14.** Parameter Recovery of 2-Level HGF with Loss Aversion Belief Trajectories


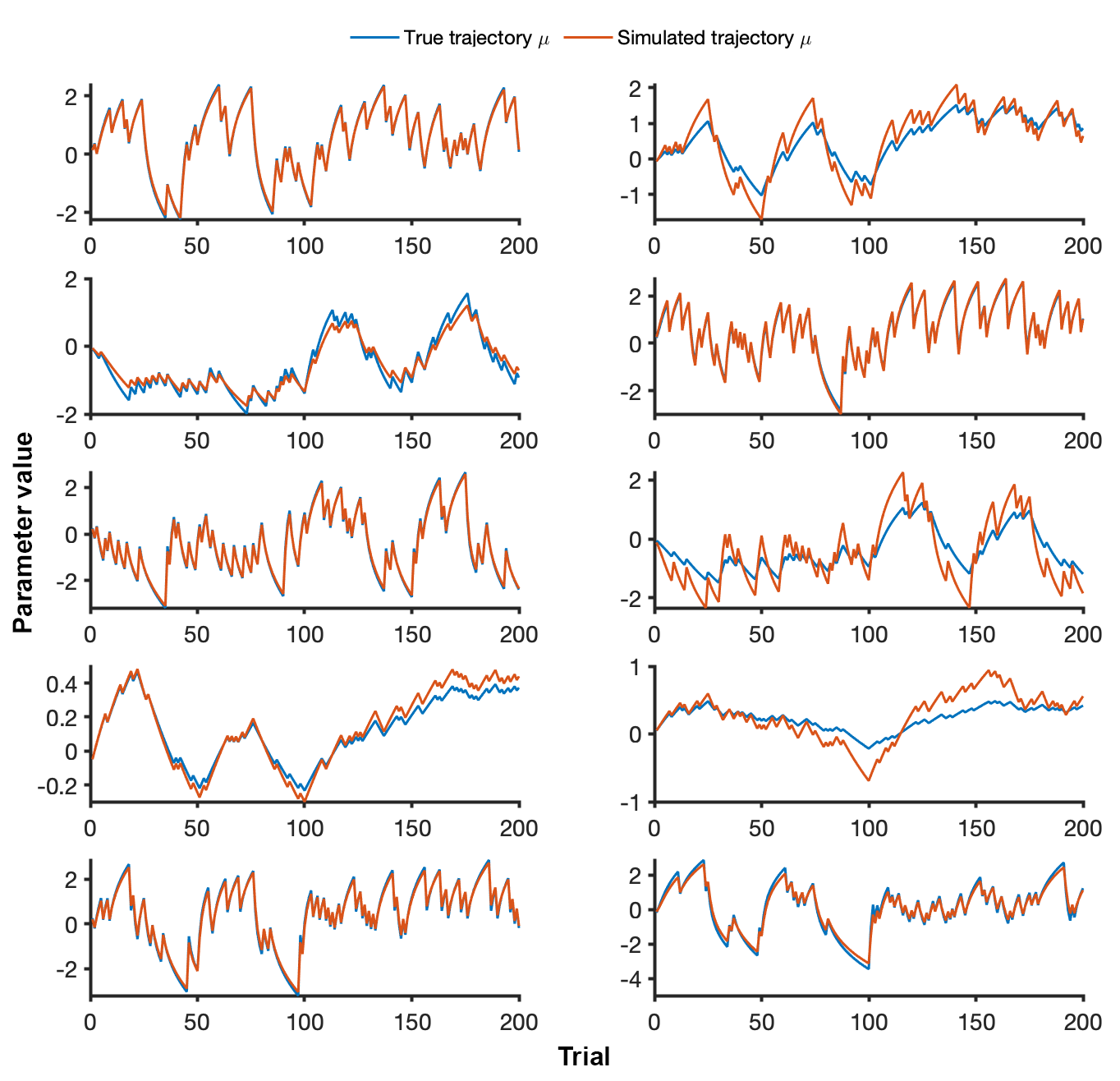


*Note*. Estimated (blue) and simulated (orange) second-level belief trajectories for 10 example participants.

**References**

1 Ramey, T. & Regier, P. S. Cognitive impairment in substance use disorders. *CNS Spectr* **24**, 102-113 (2019). <https://doi.org:10.1017/s1092852918001426>

2 Castellanos-Ryan, N. & Conrod, P. in *Cognition and Addiction* (ed Antonio Verdejo-Garcia) 91-102 (Academic Press, 2020).

3 Bechara, A. & Martin, E. M. Impaired decision making related to working memory deficits in individuals with substance addictions. *Neuropsychology* **18**, 152-162 (2004). <https://doi.org:10.1037/0894-4105.18.1.152>

4 Hakimi, N., Chou, K. P., Stewart, J. L., Paulus, M. P. & Smith, R. Computational Mechanisms of Learning and Forgetting Differentiate Affective and Substance Use Disorders. *Res Sq* (2024). <https://doi.org:10.21203/rs.3.rs-4682224/v1>

5 Wechsler, D. A standardized memory scale for clinical use. *The Journal of Psychology: Interdisciplinary and Applied* **19**, 87-95 (1945). <https://doi.org:10.1080/00223980.1945.9917223>

6 Barry, D. & Petry, N. M. Predictors of decision-making on the Iowa Gambling Task: Independent effects of lifetime history of substance use disorders and performance on the Trail Making Test. *Brain and Cognition* **66**, 243-252 (2008). <https://doi.org:https://doi.org/10.1016/j.bandc.2007.09.001>

7 Chamberlain, S. R., Odlaug, B. L., Schreiber, L. R. N. & Grant, J. E. Association between tobacco smoking and cognitive functioning in young adults. *The American Journal on Addictions* **21**, S14-S19 (2012). <https://doi.org:10.1111/j.1521-0391.2012.00290.x>

8 Kurzban, R., Duckworth, A., Kable, J. W. & Myers, J. An opportunity cost model of subjective effort and task performance. *Behav Brain Sci* **36**, 661-679 (2013). <https://doi.org:10.1017/s0140525x12003196>

9 Fortenbaugh, F. C., DeGutis, J. & Esterman, M. Recent theoretical, neural, and clinical advances in sustained attention research. *Ann N Y Acad Sci* **1396**, 70-91 (2017). <https://doi.org:10.1111/nyas.13318>

10 Allen, D. N. & Haderlie, M. M. in *The Corsini Encyclopedia of Psychology* 1-1 (2010).

11 Tombaugh, T. N. Trail Making Test A and B: Normative data stratified by age and education. *Archives of Clinical Neuropsychology* **19**, 203-214 (2004). <https://doi.org:https://doi.org/10.1016/S0887-6177(03)00039-8>

12 Smith, G. T. & Cyders, M. A. Integrating affect and impulsivity: The role of positive and negative urgency in substance use risk. *Drug and Alcohol Dependence* **163**, S3-S12 (2016). <https://doi.org:https://doi.org/10.1016/j.drugalcdep.2015.08.038>

13 Bechara, A. Decision making, impulse control and loss of willpower to resist drugs: a neurocognitive perspective. *Nature neuroscience* **8**, 1458-1463 (2005).

14 Berg, J. M., Latzman, R. D., Bliwise, N. G. & Lilienfeld, S. O. Parsing the heterogeneity of impulsivity: A meta-analytic review of the behavioral implications of the UPPS for psychopathology. *Psychological assessment* **27**, 1129 (2015).

15 Hershberger, A. R., Um, M. & Cyders, M. A. The relationship between the UPPS-P impulsive personality traits and substance use psychotherapy outcomes: A meta-analysis. *Drug and Alcohol Dependence* **178**, 408-416 (2017). <https://doi.org:https://doi.org/10.1016/j.drugalcdep.2017.05.032>

16 McCarty, K. N., Morris, D. H., Hatz, L. E. & McCarthy, D. M. Differential Associations of UPPS-P Impulsivity Traits With Alcohol Problems. *Journal of Studies on Alcohol and Drugs* **78**, 617-622 (2017). <https://doi.org:10.15288/jsad.2017.78.617>

17 Coskunpinar, A., Dir, A. L. & Cyders, M. A. Multidimensionality in Impulsivity and Alcohol Use: A Meta-Analysis Using the UPPS Model of Impulsivity. *Alcoholism: Clinical and Experimental Research* **37**, 1441-1450 (2013). <https://doi.org:https://doi.org/10.1111/acer.12131>

18 Whiteside, S. P. & Lynam, D. R. The Five Factor Model and impulsivity: using a structural model of personality to understand impulsivity. *Pers Indiv Differ* **30**, 669-689 (2001). <https://doi.org:https://doi.org/10.1016/S0191-8869(00)00064-7>

19 Patrick, C. J., Curtin, J. J. & Tellegen, A. Development and validation of a brief form of the Multidimensional Personality Questionnaire. *Psychological Assessment* **14**, 150-163 (2002). <https://doi.org:10.1037/1040-3590.14.2.150>

20 Mathys, C., Daunizeau, J., Friston, K. J. & Stephan, K. E. A Bayesian foundation for individual learning under uncertainty. *Frontiers in human neuroscience* **5**, 39 (2011).

21 Mathys, C. D., Lomakina, E. I., Daunizeau, J., Iglesias, S., Brodersen, K. H., Friston, K. J. *et al.* Uncertainty in perception and the Hierarchical Gaussian Filter. *Frontiers in Human Neuroscience* **8** (2014). <https://doi.org:10.3389/fnhum.2014.00825>

22 Blain, B. & Rutledge, R. B. Momentary subjective well-being depends on learning and not reward. *eLife* **9**, e57977 (2020). <https://doi.org:10.7554/eLife.57977>

23 Massi, B., Donahue, C. H. & Lee, D. Volatility Facilitates Value Updating in the Prefrontal Cortex. *Neuron* **99**, 598-608.e594 (2018). <https://doi.org:10.1016/j.neuron.2018.06.033>

24 Manning, C., Kilner, J., Neil, L., Karaminis, T. & Pellicano, E. Children on the autism spectrum update their behaviour in response to a volatile environment. *Developmental Science* **20**, e12435 (2017). <https://doi.org:https://doi.org/10.1111/desc.12435>

25 Behrens, T. E., Woolrich, M. W., Walton, M. E. & Rushworth, M. F. Learning the value of information in an uncertain world. *Nat Neurosci* **10**, 1214-1221 (2007). <https://doi.org:10.1038/nn1954>

26 Browning, M., Behrens, T. E., Jocham, G., O'Reilly, J. X. & Bishop, S. J. Anxious individuals have difficulty learning the causal statistics of aversive environments. *Nat Neurosci* **18**, 590-596 (2015). <https://doi.org:10.1038/nn.3961>

27 Gagne, C., Zika, O., Dayan, P. & Bishop, S. J. Impaired adaptation of learning to contingency volatility in internalizing psychopathology. *Elife* **9** (2020). <https://doi.org:10.7554/eLife.61387>

28 Hein, T. P., de Fockert, J. & Ruiz, M. H. State anxiety biases estimates of uncertainty and impairs reward learning in volatile environments. *NeuroImage* **224**, 117424 (2021). <https://doi.org:https://doi.org/10.1016/j.neuroimage.2020.117424>
